# Genetically Engineered Membrane‐Coated Nanoparticles for Enhanced Prostate‐Specific Membrane Antigen Targeting and Ferroptosis Treatment of Castration‐Resistant Prostate Cancer

**DOI:** 10.1002/advs.202401095

**Published:** 2024-07-01

**Authors:** Yu Li, Hongji Li, Keying Zhang, Chao Xu, Jingwei Wang, Zeyu Li, Yike Zhou, Shaojie Liu, Xiaolong Zhao, Zhengxuan Li, Fa Yang, Wei Hu, Yuming Jing, Peng Wu, Jingliang Zhang, Changhong Shi, Rui Zhang, Wenkai Jiang, Nianzeng Xing, Weihong Wen, Donghui Han, Weijun Qin

**Affiliations:** ^1^ Department of Urology, Xijing Hospital Air Force Medical University No.127 Western Changle Road Xi'an Shaanxi 710032 China; ^2^ State Key Laboratory of Oral Maxillofacial Reconstruction and Regeneration National Clinical Research Center for Oral Diseases Shaanxi Key Laboratory of Stomatology Department of Operative Dentistry and Endodontics School of Stomatology Air Force Medical University No.145 Western Changle Road Xi'an Shaanxi 710032 China; ^3^ Department of Medicine Chemistry and Pharmaceutical Analysis School of Pharmacy Air Force Medical University No.169 Western Changle Road Xi'an Shaanxi 710032 China; ^4^ Division of Cancer Biology Laboratory Animal Center Air Force Medical University No.169 Western Changle Road Xi'an Shaanxi 710032 China; ^5^ The State Key Laboratory of Cancer Biology Department of Immunology Air Force Medical University No.169 Western Changle Road Xi'an Shaanxi 710032 China; ^6^ State Key Laboratory of Molecular Oncology National Cancer Center National Clinical Research Center for Cancer Department of Urology Cancer Hospital Chinese Academy of Medical Sciences and Peking Union Medical College Beijing 100021 China; ^7^ Institute of Medical Research Northwestern Polytechnical University Xi'an Shaanxi 710072 China; ^8^ National Translational Science Center for Molecular Medicine Department of Cell Biology Air Force Medical University No.169 Western Changle Road Xi'an Shaanxi 710032 China

**Keywords:** castration‐resistant prostate cancer, ferroptosis, macrophage membrane‐coated nanoparticles, PSMA, tumor targeting therapy

## Abstract

Conventional androgen deprivation therapy (ADT) targets the androgen receptor (AR) inhibiting prostate cancer (PCa) progression; however, it can eventually lead to recurrence as castration‐resistant PCa (CRPC), which has high mortality rates and lacks effective treatment modalities. The study confirms the presence of high glutathione peroxidase 4 (GPX4) expression, a key regulator of ferroptosis (i.e., iron‐dependent program cell death) in CRPC cells. Therefore, inducing ferroptosis in CRPC cells might be an effective therapeutic modality for CRPC. However, nonspecific uptake of ferroptosis inducers can result in undesirable cytotoxicity in major organs. Thus, to precisely induce ferroptosis in CRPC cells, a genetic engineering strategy is proposed to embed a prostate‐specific membrane antigen (PSMA)‐targeting antibody fragment (gy1) in the macrophage membrane, which is then coated onto mesoporous polydopamine (MPDA) nanoparticles to produce a biomimetic nanoplatform. The results indicate that the membrane‐coated nanoparticles (MNPs) exhibit high specificity and affinity toward CRPC cells. On further encapsulation with the ferroptosis inducers RSL3 and iron ions, MPDA/Fe/RSL3@M‐gy1 demonstrates superior synergistic effects in highly targeted ferroptosis therapy eliciting significant therapeutic efficacy against CRPC tumor growth and bone metastasis without increased cytotoxicity. In conclusion, a new therapeutic strategy is reported for the PSMA‐specific, CRPC‐targeting platform for ferroptosis induction with increased efficacy and safety.

## Graphical abstract

The study confirms the high GPX4 expression, a key regulator of ferroptosis in CRPC cells. To precisely induce ferroptosis in CRPC, a genetic engineering strategy is proposed to embed a PSMA‐targeting antibody fragment (gy1) in the macrophage membrane and produce the membrane‐coated nanoparticles encapsulating with the ferroptosis inducers. The PSMA‐specific, CRPC‐targeting platform for ferroptosis induction demonstrates increased efficacy and safety.

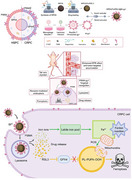

## 1 Introduction

Prostate cancer (PCa) remains the second most commonly diagnosed malignant cancer among men worldwide.^[^ ^1^ ^]^ In the United States, PCa accounts for 34 500 deaths annually, almost from advanced disease.^[^ ^2^ ^]^ The primary treatment for men with advanced PCa is androgen deprivation therapy (ADT), in which androgen receptor (AR) signaling is inhibited. Despite initial ADT response being effective, it ultimately fails in most patients with PCa, leading to castration‐resistant PCa (CRPC) development, reducing the 5‐year relative survival rate to 30%.^[^ ^3^ ^]^ Currently, first‐line drugs for CRPC, such as second‐generation androgen inhibitors (e.g., enzalutamide, abiraterone, and apalutamide), have demonstrated considerable effectiveness. However, drug resistance and low responsiveness have limited the application of these drugs;^[^ ^4^ ^]^ this is because CRPC cells have many complex resistance mechanisms, including AR amplification, mutation, or splice variants, that aid them in maintaining their AR function in low‐androgen environments.^[^ ^5^ ^]^ Meanwhile, AR inhibitors may hence dedifferentiation to a hybrid epithelial and mesenchymal, stem‐like state and neuroendocrine PCa leading to lineage plasticity and metastasis, which promotes resistance to AR inhibition therapy.^[^ ^6^ ^]^ Therefore, exploring novel therapeutic approaches independent of the AR signaling axis for CRPC treatment is crucial.

Ferroptosis, a recently identified form of programmed cell death, is characterized by the accumulation of iron‐dependent lethal lipid hydroperoxide (LPO), including reactive oxygen species (ROS) and polyunsaturated fatty acids (PUFAs).^[^ ^7^ ^]^ Iron ions (Fe^2+^/Fe^3+^) can react with peroxides in cells and generate ROS through the Fenton reaction.^[^ ^8^ ^]^ AR inhibitors can reprogram the metabolic state of PCa, leading to an accumulation of lipids, which supply energy for bioenergetic processes and cell proliferation. This increase in the concentrations of lipids, particularly PUFAs, can enhance cell membrane fluidity and lipid peroxidation. Thus, AR inhibitor–resistant CRPC cells may acquire the persister cell phenotype which is characterized by increased dedifferentiation marker expression, enhanced lipid peroxidation, and ferroptosis sensitivity, suggesting that ferroptosis induction may lead to considerable advantages in the treatment of AR inhibitor–resistant CRPC.^[^ ^9^ ^]^

Ferroptosis activation involves a series of complexes that reciprocally regulate redox homeostasis, lipid metabolism, and iron metabolism,^[^ ^10^ ^]^ and researchers have developed ferroptosis‐inducing drugs, acting on the key molecules of ferroptosis such as solute carrier family 7, member 11 (SLC7A11), glutathione peroxidase 4 (GPX4), and ferroptosis suppressor protein 1 (FSP1).^[^ ^8^, ^11^ ^]^ However, ferroptosis activations are physiological processes equally important for maintaining normal cell homeostasis and physiological functions, and most small‐molecule ferroptosis inducers or iron ion drugs are highly hydrophobic with low biological stability; and the nonspecific uptake of these ferroptosis inducers may cause undesirable cytotoxicity to major organs such as the heart, kidneys, and central nervous system, elevating the associated health risks.^[^ ^12^ ^]^ Therefore, precisely inducing ferroptosis in CRPC tumor sites may prevent the cytotoxic side effects of ferroptosis.

Recent breakthroughs in nanotechnology have yielded versatile therapeutic nanoplatforms that can overcome the limitations of conventional therapeutic drugs and improve the balance between therapeutic efficacy and adverse effects.^[^ ^13^ ^]^ Various biomimetic nanoformulations have been fabricated using natural cell membranes derived from erythrocytes,^[^ ^14^ ^]^ immune cells,^[^ ^15^ ^]^ platelets,^[^ ^16^ ^]^ cancer cells,^[^ ^17^ ^]^ and stem cells^[^ ^18^ ^]^ for customized biomedical applications. Cell membrane–camouflaged nanomaterials, which can be used to create biomimetic nanoparticles, have garnered considerable attention because of their unique properties inherited from source cells.^[^ ^19^ ^]^ The targeting ability of the biomimetic nanoparticles is often mediated by proteins expressed on the source cells, which endows nanoparticles with the capability to specifically interact with various disease substrates but most membrane‐based nanomedicines only use the natural homing property of cell membranes to migrate toward tumor sites, leading to reductions in targeting ability and migration efficiency. Some researchers have attempted to introduce exogenous tumor‐targeting moieties (e.g., small molecules, aptamers, peptides, and antibodies) on cell membrane surfaces via lipidized ligand insertion or chemical coupling, however, many uncontrollable factors, including tedious modification processes, unstable coupling linkers, prevailing membrane protein denaturation, and unpredictable binding sites, and unreasonable ligand packing density, often negatively impact the targeting efficiency, posing additional challenges toward the translation of nanomedicines into clinical practice.^[^ ^20^ ^]^ Development of approaches that improve the stability and targeting efficiency of cell membrane–coated nanomedicine for CRPC therapy is highly warranted.

Prostate‐specific membrane antigen (PSMA), a transmembrane glutamate carboxypeptidase, is strongly expressed on PCa cells, with its expression level in CRPC being much higher than that in other PCa types.^[^ ^21^ ^]^ Hence, PSMA is a well‐established target for CRPC diagnosis and treatment.^[^ ^22^ ^]^ We previously reported that a PSMA extracellular domain‐specific single‐chain antibody fragment (scFv), termed gy1, obtained from a large yeast‐display naive human scFv library; gy1 could specifically bind to PSMA with a high binding affinity and become efficiently internalized into PSMA^+^ PCa cells.^[^ ^23^ ^]^ Herein, rather than relying on postfabrication approaches to incorporate additional functionality, in this study, we propose a novel genetic engineering strategy to stably transmembrane (TM)‐express a tumor‐specific ligand on macrophages to increase the active targeting and therapeutic efficacies of macrophage membrane–based nanomedicines. We also first report the design of a composite therapeutic biomimetic nanoplatform, based on our findings with gy1, which combines actively PSMA‐targeting features and ferroptosis‐inducing drug delivery. To increase its biocompatibility and drug‐loading capacity (DLC), we used mesoporous polydopamine (MPDA) for the drug‐loaded core; then, iron ions and ferroptosis inducer RSL3 were loaded through π–π stacking, hydrophobic interaction, or both to obtain MPDA/Fe/RSL3. We further TM‐expressed gy1 on macrophages through lentiviral infection and harvested the engineered membrane (M‐gy1) to encapsulate the MPDA/Fe/RSL3 nanoparticles to eventually obtain MPDA/Fe/RSL3@M‐gy1. These membrane‐coated nanoparticles (MNPs) were endowed with the targeting ability to PSMA and transferred into CRPC cell endosomes and lysosomes through receptor‐mediated endocytosis and activated by the lysosomal acidity (pH = 5), triggering iron ion and RSL3 release. At the tumor site, the released Fe^3+^ and Fe^2+^ may catalyze the reactions to produce LPOs, resulting in ferroptosis, specifically in the H_2_O_2_‐overloaded cancer cells.^[^ ^24^ ^]^ Meanwhile, RSL3 lead to GPX4 inhibition, which then leads to ferroptosis induction in cancer cells.^[^ ^25^ ^]^ In general, this study reveals a new therapeutic strategy using a PSMA‐specific, CRPC‐targeting platform of ferroptosis induction with increased efficacy and safety; our results may also expand the current of ferroptosis in the CRPC landscape.

## 2 Results and Discussion

### 2.1 Association of GPX4 Expression with Progression to CRPC

**Figure 1:**
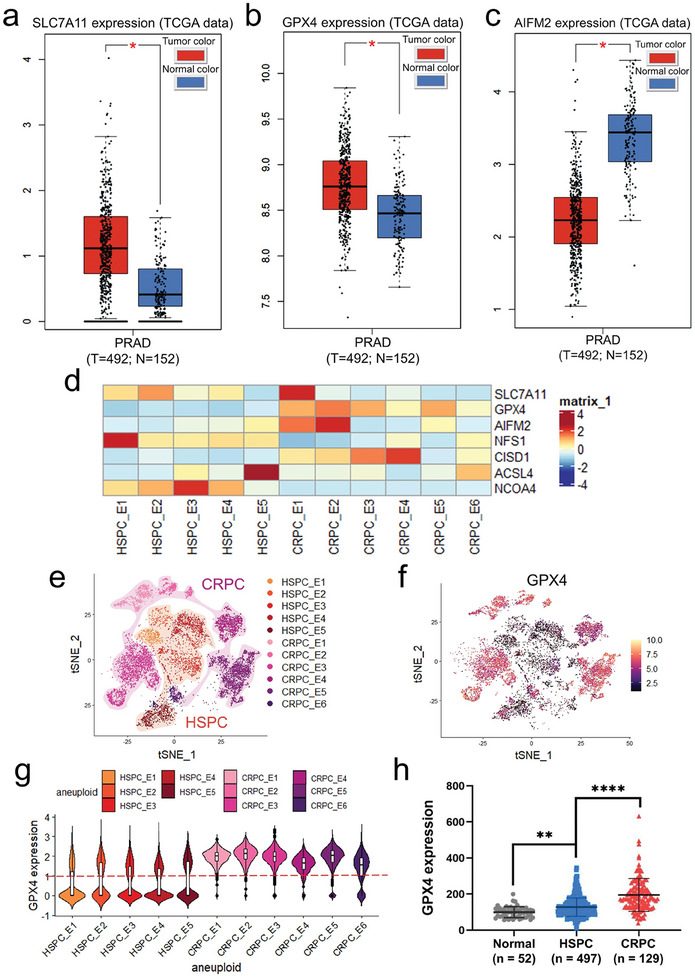
Association of ferroptosis related gene expression with progression to CRPC. a) Expression of *SLC7A11* in PCa tissues and normal tissues in the GEPIA database. The bar height represents the median *SLC7A11* expression. b) Expression of *GPX4* in PCa tissues and normal tissues in the GEPIA database. c) Expression of *AIFM2* (FSP1) in PCa tissues and normal tissues in the GEPIA database. d) Heatmap showing the expression levels of selected genes related to ferroptosis among epithelial subclusters. e) UMAP plot of subclusters of aneuploid epithelial cells. Cells derived from patients with HSPC or CRPC were marked using a differential color scheme. f) UMAP representation of GPX4 expression. g) Violin plots for GPX4 expression (*y* axis) in each epithelial subpopulation (*x* axis). h) Comparison of GPX4 expression levels in PCa adjacent, HSPC, and CRPC tissues in TCGA and the cBioPortal databases. *p < 0.05, **p < 0.01, ****p < 0.0001.

In order to identify the critical genes involved in ferroptosis after resistance to ADT, we first assessed the expression of ferroptosis‐related genes based on PCa and nonneoplastic samples in the GEPIA database (http://gepia.cancer‐pku.cn/). Our results indicated that *SLC7A11* and *GPX4* were significantly higher in tumor tissues than in normal prostate tissues (**Figure** **1**a,b) and the opposite result was observed for *AIFM2* (FSP1) (Figure 1c). To furtherly confirm differential ferroptosis‐related genes expression between hormone‐sensitive prostate cancer (HSPC) and CRPC tumor cells, we performed an integrated single‐cell sequencing analysis based on the HSPC dataset GEO176031^[^ ^26^ ^]^ and the CPRC dataset GEO137829.^[^ ^27^ ^]^ After applying a merging and filtering process, we deemed a total of 29 016 epithelial cells qualified for subsequent analyses (Figures S1 and S2, Supporting Information). We assessed the expression level of ferroptosis‐related proteins including SLC7A11, GPX4, AIFM2, cysteine desulfurase (NFS1), CDGSH iron sulfur domain protein 1 (CISD1), Acyl‐CoA synthetase long chain family member 4 (ACSL4), and nuclear receptor coactivator 4 (NCOA4). The results validated GPX4 as an excellent target for ferroptosis inhibition in CRPC (Figure 1d). After performing copy number karyotyping of tumors (copyKAT)^[^ ^28^ ^]^ to select 12.485 carcinoma cells (Figure S3, Supporting Information), we further identified five epithelial subclusters for HSPC and six subsets for CRPC in the integrated dataset (Figure 1e). Notably, GPX4 was considerably expressed in the epithelium derived from CRPC compared with HSPC (Figure 1f,g). To provide additional evidence for increased *GPX4* mRNA expression in patients with CRPC, we performed integration and analysis of bulk‐sequencing datasets obtained from The Cancer Genome Atlas (TCGA) and SU2C.^[^ ^29^ ^]^ After batch processing, our findings revealed elevated *GPX4* expression in the prostate tumors compared with the adjacent tissues. Furthermore, further augmentation of *GPX4* and *FOLH1* (PSMA) expression in CRPC indicated a potential association between disease progression, androgen resistance, and enhanced resistance to ferroptosis within the tumor microenvironment (Figure 1g; Figure S4, Supporting Information). Patients with high *GPX4* expression had shorter disease‐free survival than patients with low *GPX4* expression in PCa datasets in the GEPIA database (Figure S5, Supporting Information), indicating that *GPX4* expression is higher in recurrent PCa tissues, including CRPC.

**Figure 2:**
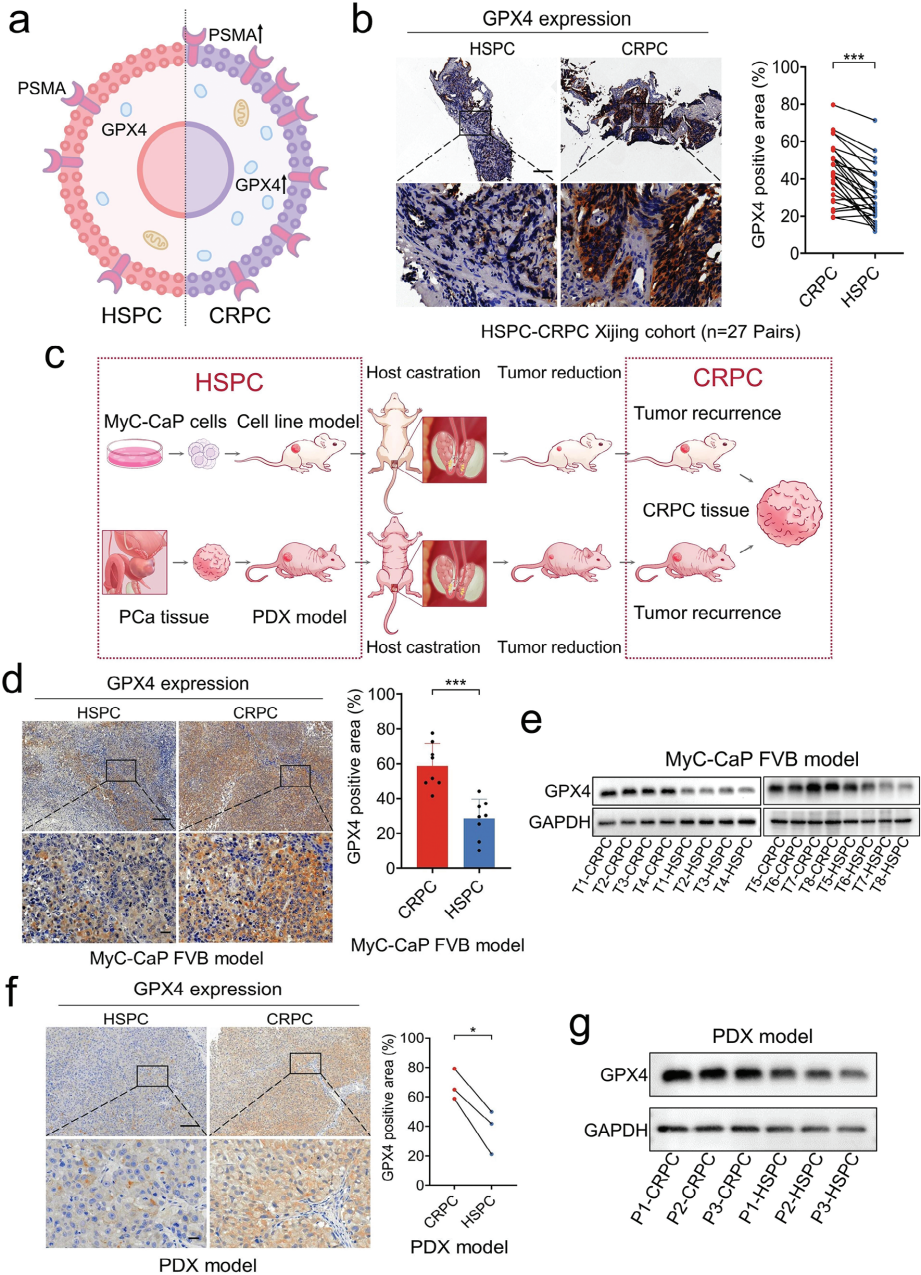
Association of GPX4 expression with progression to CRPC. a) Significant progression of CRPC indicated by GPX4 and PSMA expression levels. b) Representative IHC staining for GPX4 expression in PCa tumor tissues at HSPC and CRPC stages and matched‐pair analysis of GPX4 expression levels in 27 PCa tissues at HSPC and CRPC stages. Scale bars: 200 and 50 µm. c) Surgical castration performed in mice bearing subcutaneous PDX or MyC‐CaP tumor tissues to induce and establish mouse models of CRPC. d) Representative IHC staining for difference in GPX4 expression in HSPC and CRPC MyC‐CaP FVB model tissues and the comparison between GPX4 expression levels in HSPC and CRPC MyC‐CaP FVB model tissue. Scale bars: 200 and 50 µm. e) Western blots for GPX4 expression levels in HSPC and CRPC MyC‐CaP FVB models. f) Representative IHC staining for different GPX4 expression in HSPC and CRPC PDX model tissue and matched‐pair analysis of GPX4 expression levels in three PDX model tissues at HSPC and CRPC stages. Scale bars: 200 and 50 µm. g) Western blot for GPX4 expression levels in HSPC and CRPC PDX models. *p < 0.05, ***p < 0.001.

The current and previous results indicated that GPX4 expression increases significantly in therapy‐resistant states of PCa (e.g., CRPC; **Figure** **2**a); thus, GPX4 inhibition might be an effective CRPC treatment target. To examine the protein expression pattern of GPX4 in PCa further, we performed immunohistochemical (IHC) staining assays for 100 cases of PCa and 50 cases of nonneoplastic tissues. As shown in Figure S6 (Supporting Information), this result was consistent with that of the GEPIA analysis: GPX4 expression was higher in the PCa tissues. On the basis of the clinicopathological data of the corresponding patients, we analyzed the correlation of PCa clinical stage with GPX4 expression; the results indicated that GPX4 expression increased significantly with clinical stage progression (Figure S7a, Supporting Information) but not with that of the pathological grade (Gleason score; Figure S7b, Supporting Information). Furthermore, in the 27‐patient cohort from Xijing Hospital, we performed IHC staining for GPX4 expression in both hormone‐sensitive PCa (HSPC) and CRPC tissues (Figure 2b) and PSMA as a marker (Figure S8, Supporting Information) to identify tumor tissue distribution. The result of our paired statistical analysis of the GPX4‐positive area suggested that GPX4 expression was significantly higher in CRPC than in HSPC (Figure 2b).

Based on the HSPC mouse‐derived cell line MyC‐CaP, we constructed subcutaneous HSPC tumor models in FVB mice. We then applied surgical castration to these HSPC models and simultaneously monitored their serum testosterone levels and tumor sizes. Tumor recurrence with low testosterone level was considered to indicate that our HSPC models developed CRPC. After surgical castration, we obtained eight CRPC models (Figure 2c). Based on the tumor tissues of models, GPX4 expression was confirmed in HSPC and CRPC tissues through quantitative reverse transcription polymerase chain reaction (qRT‐PCR; Figure S9a, Supporting Information), IHC staining (Figure 2d) and Western blotting (Figure 2e). The results indicated that GPX4 expression was significantly higher in the CRPC tissues than in the HSPC tissues. Next, three patient‐derived xenograft (PDX) models of HSPC were constructed and developed into those of CRPC through castration (Figure 2c). The HSPC and CRPC models of PDX demonstrated GPX4 expression consistent with that in our MyC‐CaP cell line models (Figure 2f,g; Figure S9b, Supporting Information).

Studies have reported that GPX4 dependence exists across diverse therapy‐resistant states of cancer cells and that cell death induced by lipid peroxidase pathway inhibition is a feature of therapy‐resistant cancer.^[^ ^30^ ^]^ Next, we performed Cell Counting Kit‐8 (CCK‐8) assay to assess RSL3‐induced death in the CRPC cell lines 22RV1 and C42; the results demonstrated that RSL3 was significantly toxic to C42 and 22RV1 cells (50% inhibiting concentration, IC50 = 2.169 and 1.118 µm, respectively; Figure S10a,b, Supporting Information). This result is consistent with that of Ghoochani et al.^[^ ^31^ ^]^ Furthermore, to verify whether RSL3 is toxic to normal cells, we coincubated different concentrations of RSL3 with the normal human liver cell line MIHA and the human renal cortex proximal convoluted tubule epithelial cell line HK‐2. Our CCK‐8 assay results indicated that RSL3 is cytotoxic toward both MIHA and HK‐2 cells (IC50 = 2.169 and 1.118 µm, respectively; Figure S10c,d, Supporting Information). Therefore, although GPX4 can be used as an effective therapeutic target for CRPC, direct cytotoxicity of its inhibitor RSL3 might kill normal tissue cells and cause serious toxic side effects. We also performed the iron ions‐related (FeSO_4_) CCK‐8 assay, and found that while it inhibited CRPC cells (IC50 = 1066 µm and 804.6 µm, respectively; Figure S11a,b, Supporting Information), it still caused indiscriminately attacks on normal liver and renal cells lacking specific tumor targeting ability (IC50 = 380.2 µm and 345.8 µm, respectively; Figure S11c,d, Supporting Information). In vivo, we injected RSL3 and iron ions (FeSO_4_) into the 22RV1‐bearing mice by tail vein administration. We observed a sharp decline in body weight within two weeks, necessitating termination of the experiment according to animal ethics protocols (Figure S12, Supporting Information). The above in vitro and in vivo results indicate that treatment with these two drugs alone would cause indiscriminate killing to normal tissue and cells leading to significant biological toxicity. As such, a new drug delivery carrier, which can precisely deliver the drug to CRPC tumor tissues and provide biological safety, should be designed and constructed.

### 2.2 PSMA Targeted MNPs Fabrication and Characterization

**Figure 3:**
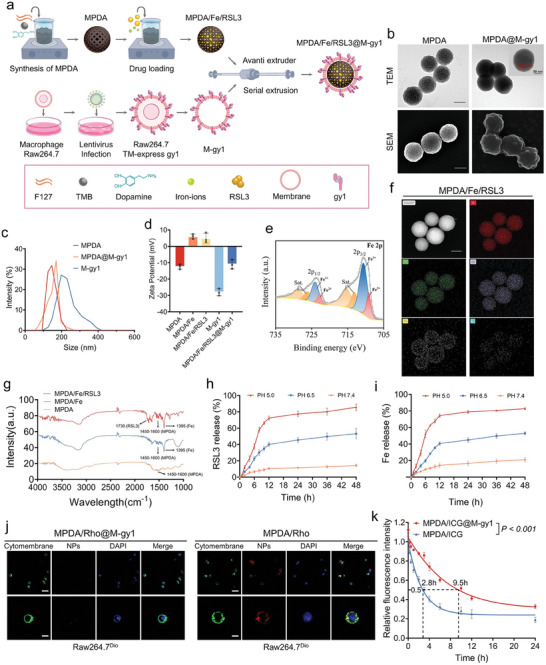
Fabrication and characterization of MNPs. a) Schematic of MPDA/Fe/RSL3@M‐gy1 preparation process. b) TEM and SEM images of MPDA and MPDA@M‐gy1. Scale bar: 100 nm. c) Size distributions of MPDA, MPDA@M‐gy1, and M‐gy1. d) Zeta potentials of MPDA, MPDA/Fe, MPDA/Fe/RSL3, M‐gy1, and MPDA/Fe/RSL3@M‐gy1. e) Fe 2p XPS spectra of MPDA/Fe/RSL3@M‐gy1. f) STEM image of MPDA/Fe/RSL3, and the corresponding EDS analysis results. g) FTIR spectra of MPDA, MPDA/Fe, and MPDA/Fe/RSL3. h) Percentage of RSL3 released from MNPs in solutions with different pH values for 48 h. i) Percentage of Fe ions released from the MNPs in solutions with different pH values for 48 h. j) Immune evasion ability of MNPs was evaluated through CLSM. Raw264.7 cells labeled with 3,3′‐dioctadecyloxacarbocyanine perchlorate (Dio; green), and MPDA/Rho and MPDA/Rho@M‐gy1 labeled with preloaded rhodamine B (red). Scale bars: 50 and 10 µm. k) Circulatory half‐life of MPDA/ICG@M‐gy1 detected on a fluorescent microplate reader. MPDA/ICG was used as the control. Data are shown as means ± standard deviations from at least three independent experiments.

Because of the nondifferential toxicity of ferroptosis inducers, we need to find a PCa specific target. Considering the importance of PSMA in PCa treatment and the high affinity of gy1 toward PSMA,^[^ ^22^, ^23^ ^]^ a macrophage‐based platform with enhanced PSMA‐targeting ability might be obtained through the integration of gy1 with macrophages. To confirm this, we genetically engineered Raw264.7 cells to stably TM‐express gy1 through lentiviral infection and obtain M‐gy1. The lentiviral vector used and preparative procedures of M‐gy1 are schematically displayed in **Figures** **3**a and S13a (Supporting Information). In brief, the extracellular segment of gy1 acting as the PSMA‐targeting moiety and cytoplasmic domain of enhanced green fluorescent protein (eGFP), as a reporter, were anchored onto the macrophage membrane via a rational intracellular protein synthesis process by using the CD8 hinge domain and CD8 TM region. High‐level (nearly 100%) expression of the gy1‐eGFP fusion protein was detected through confocal laser scanning microscopy (CLSM), flow cytometry (FCM), and Western blotting, which confirmed that efficient expression of gy1 was on the macrophage membrane (Figure S13b–d, Supporting Information). This is a prerequisite for endowing macrophage membrane–based nanomedicines with improved PSMA targetability.

Subsequently, we synthesized our biopolymer nanoplatform, as illustrated in Figure 3a. We first prepared MPDA, partly based on previously reported methods.^[^ ^32^ ^]^ Our transmission electron microscopy (TEM) and the scanning electron microscopy (SEM) observations indicated that MPDA had a clear, uniform mesoporous spherical structure with a radially arranged mesochannel (Figure 3b). Brunauer–Emmett–Teller (BET) analysis indicated that the adsorption and desorption average pore diameters were 8.85 and 9.56 nm, respectively, based on the Figure S14a,b (Supporting Information). We further extracted the macrophage membrane and prepared the macrophage membrane–camouflaged MPDA nanoparticles (designated as MPDA@M‐gy1) by using a previously reported serial extrusion method^[^ ^33^ ^]^ (Figure 3a). Compared with bare MPDA, MPDA@M‐gy1 had a clear core–shell structure (Figure 3b), indicating the presence of a unilamellar cell membrane coating on the nanoparticles. Moreover, the outer shell thickness was ≈12.56 nm, and MPDA's mesopore structure became blurred after it was covered by the cell membrane, indicating that the macrophage membrane provided prominent coverage over MPDA (Figure 3b). According to the nano measurer, the average diameter of MPDA@M‐gy1 was 171.8 ± 25.89 nm—slightly greater than that of bare MPDA (155.6 ± 26.53 nm; Figure S14c, Supporting Information); the dynamic light scattering results confirmed that the nanoparticle size after macrophage membrane wrapping was slightly larger than that of bare MPDA (Figure 3c). Then, the colloidal stability of MPDA@M‐gy1 was examined by measuring the size changes over time (Figure S14d, Supporting Information). Additionally, MPDA/Fe/RSL3@M‐gy1 exhibited higher stability with negligible size change in different solvents over 7 days than the MPDA/Fe/RSL3 without encapsulating the cell membrane (Figure S14e, Supporting Information).

Because of the abundant aromatic rings and catechol groups and the mesoporous structure, MPDA demonstrated high loading capacity for both RSL3 and iron ions.^[^ ^34^ ^]^ The zeta potential of MPDA/Fe/RSL3 changed from −12.45 ± 1.51 to 5.007 ± 1.74 mV after iron ions and RSL3 were loaded and then to −10.99 ± 2.821 mV, approaching the zeta potential value of pure macrophage membrane (−28.04 ± 1.238 mV; Figure 3d). Our X‐ray photoelectron spectroscopy (XPS) results further suggested that both Fe^2+^ and Fe^3+^ were present throughout the preparation process, indicating that iron ions were successfully chelated in MPDA (Figure 3e). Scanning transmission electron microscopy (STEM)‐based elemental mapping results demonstrated that the coordinated iron ions and RSL3 (in the form of its characteristic element chlorine) were homogeneously distributed on the nanoparticle surface (Figure 3f), whereas RSL3 incorporation was confirmed through UV–visible (UV‐vis) spectroscopy (Figure S15, Supporting Information). Moreover, the stepwise modification process was further monitored and established by using Fourier‐transform infrared (FTIR) spectra (Figure 3g). The DLC and encapsulation efficiency (EE) of RSL3 in MPDA/Fe/RSL3 were quantified by reading the absorbance of saturated absorption spectroscopy (SAS) at 285 nm. When the RSL3‐to‐MPDA nanoparticle concentration ratio was 4:1, the drug loading and encapsulation rate appeared at the intersection of the curve; the concentration ratio of 4:1 was thus determined as the optimal drug‐loading parameter (Figure S16a, Supporting Information). The iron content of products was then analyzed through inductively coupled plasma mass spectrometry (ICP‐MS). The iron ion contents in nanoparticles with different mass ratios were measured, and the highest chelation efficiency was obtained at a MPDA: FeSO_4_·7H_2_O mass ratio of 1:100 (Figure S16b, Supporting Information). Changes to endogenous parameters such as pH can provide controllable tumor‐targeted drug release. Notably, RSL3–MPDA π–π stacking interactions and iron ion–catechol coordination are 0 intrinsically susceptible to acidity.^[^ ^35^ ^]^ Here, an acidic pH of 5.0 substantially enhanced RSL3 release: accumulated RSL3 release was ≈70% after 12 h, and similar release patterns were observed for iron ions (Figure 3h,i).

### 2.3 In Vitro and In Vivo Immune Evasion Capability of MNPs

Nanoparticles camouflaged by biological cell membranes have recently been the focus of attention due to their prolonged circulation in vivo.^[^ ^36^ ^]^ Their innate self‐recognition features optimize their dynamic properties by camouflaging the contents to evade the elimination of the mononuclear phagocyte system (MPS) and the immunological surveillance.^[^ ^37^ ^]^ After MNPs fabrication, we conducted multiple experiments to verify whether the M‐gy1 membrane coating endowed MNPs with the capacity to avoid immune clearance. Rhodamine B (Rho)‐loaded MNPs (MPDA/Rho@M‐gy1) and Rho‐loaded MPDA (MPDA/Rho) were fabricated, and their macrophage‐mediated phagocytosis abilities were compared. Our CLSM analysis (Figure 3j; Figure S17a, Supporting Information) demonstrated that MPDA/Rho@M‐gy1 escaped phagocytosis by macrophages more effectively than MPDA/Rho. FCM measurement further confirmed that Raw264.7 cells phagocytized more MPDA/Rho than MPDA/Rho@M‐gy1 (Figure S17b, Supporting Information). This result confirmed that the M‐gy1 membrane coating conferred MNPs with enhanced immune evasion capability, potentially prolonging the retention time of MNPs in blood circulation and improving its tumor targeting ability.

Considering the effectiveness noted in our in vitro results, we next assessed MNP biodistribution in xenograft mice. Indocyanine green (ICG)‐loaded MNPs (designated as MPDA/ICG@M‐gy1) were first intravenously injected into normal BALB/c nude mice, with ICG‐loaded MPDA (MPDA/ICG) used as the control. Subsequently, 20 µL of blood was collected from the mice at different time points, and the fluorescence intensity of ICG in plasma was measured. As depicted in Figure 3k, the circulatory half‐life of MPDA/ICG@M‐gy1 was significantly extended to 9.5 h, much higher than that of MPDA/ICG (2.8 h)—indicating that macrophage membrane coating can prevent MNPs from being cleared from the blood. We also acquired in vivo fluorescence images of normal mice who were intravenously injected with MPDA/ICG@M‐gy1 or MPDA/ICG. MPDA/ICG demonstrated considerable accumulation in the liver and spleen (owing to clearance by the MPS); in contrast, MPDA/ICG@M‐gy1 appeared to have effectively avoided MPS recognition and elimination (Figure S17c, Supporting Information).

### 2.4 In Vitro and In Vivo CRPC Cell–Targeting and Internalization Ability of MNPs

**Figure 4:**
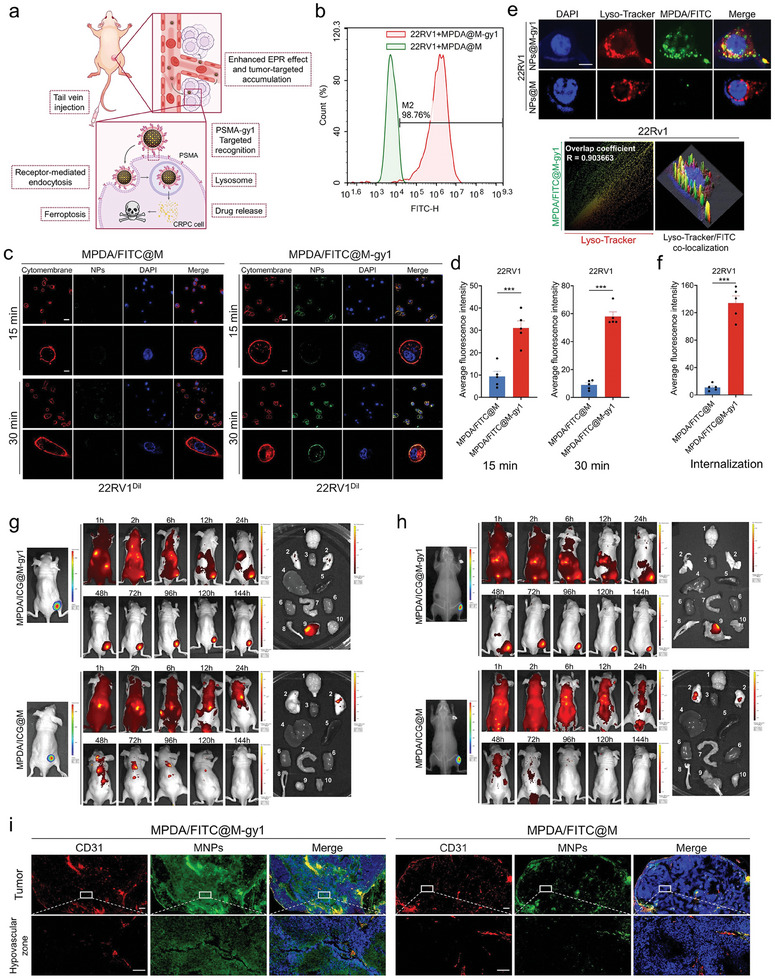
CRPC cell–targeting and internalization ability of MNPs in vitro and in vivo. a) Schematic of PSMA‐targeting ability and tumor‐specific binding and internalization of MNPs. b) Binding efficiency of MNPs to 22RV1 cells detected through FCM. MNPs without TM‐expressed gy1 were used as the control. c) Specific binding of MNPs to 22RV1 cells, evaluated through CLSM. MNPs without TM‐expressed gy1 were used as the control. MNPs were labeled with FITC (green). Cytomembranes were stained with 1,1′‐dioctadecyl‐3,3,3′,3′‐tetramethylindocarbocyanine perchlorate (Dil; red). Scale bars: 50 and 10 µm. d) Quantitative analysis of the binding average green fluorescence patterns intensity of MPDA/FITC@M‐gy1 and MPDA/FITC@M at 15 min and 30 min. e) Specific internalization of MNPs into 22RV1 cells, evaluated through CLSM. MNPs without TM‐expressed gy1 cells were used as the control. MNPs were labeled with FITC (green). Lysosomes were stained with Lyso‐Tracker (red). Scale bar: 10 µm. f) Quantitative analysis of the internalized average green fluorescence patterns intensity of MPDA/FITC@M‐gy1 and MPDA/FITC@M. g) Tumor recognition capabilities of MNPs in luciferase‐expressing 22RV1 subcutaneous tumor‐bearing mice. Bioluminescence was noted in the images of the tumor site and ex vivo tumor 144 h after injection of MNPs. h) Tumor recognition capabilities of MNPs in luciferase‐expressing 22RV1 bone metastasis tumor‐bearing mice. Bioluminescence was noted in the images of the tumor site and ex vivo tumor 144 h after injection of MNPs. i) Fluorescence localization of MNPs in 22RV1 subcutaneous tumor sections. MNPs were tracked with FITC. White squares denote hypovascular zones. Scale bars: 500 and 100 µm. Data are presented as means ± standard deviations from at least three independent experiments. ***p < 0.001.

After successful MNP fabrication, we investigated whether MNPs with TM‐expressed gy1 inherit the prominent PSMA‐specific ability and CRPC‐targeting internalization of gy1 (**Figure** **4**a). MNPs with or without TM‐expressed gy1 were incubated with PSMA‐positive CRPC cells (22RV1 and C42), and their binding affinity to PSMA was analyzed through flow cytometry. The results demonstrated that MNPs with TM‐expressed gy1 but not MNPs without TM‐expressed gy1 could specifically bind to 22RV1 and C42 cells (Figure 4b; Figure S18a, Supporting Information), probably because of their higher binding efficiency to PSMA in CRPC cells. Our CLSM results indicated that MNPs loaded with fluorescein isothiocyanate (FITC) and TM‐expressed gy1 could rapidly bind to 22RV1 and C42 cells within 15 and 30 min. The green fluorescence signal was significantly stronger in the MPDA/FITC@M‐gy1 group than in the control group (Figure 4c,d; Figure S18b,c, Supporting Information). Furthermore, due to the specific binding of gy1 to PSMA, the MNPs with TM‐expressed gy1 could be readily endocytosed by 22RV1 and C42 cells within 2 h, followed by effective internalization into lysosomes. Together, these results indicate that TM‐expressed gy1 enhanced MNPs’ PSMA targeting and cellular uptake (Figure 4e,f; Figure S18d,e, Supporting Information). We further co‐incubated excess PSMA single‐chain antibody (gy1) with 22V1 cells followed by the addition of MPDA/FITC@M‐gy1. FCM analysis of the green fluorescence intensity in each group (blank control, MPDA/FITC@M‐gy1, and gy1+MPDA/FITC@M‐gy1) confirmed a significant reduction in the targeting binding ability of the membrane‐coated nanoparticles to 22RV1 cells pre‐treated with the single‐chain antibodies (Figure S19, Supporting Information).

Although it has good targeting ability in vitro, it is still unclear whether it can stably target tumor tissue in the complex circulating environment in vivo. After confirming the prominent CRPC cell–targeting performance of MNPs with TM‐expressed gy1, we assessed the biodistribution and targeting ability in CRPC xenograft mice. MPDA/ICG@M‐gy1 or MPDA/ICG@M were intravenously injected into 22RV1 subcutaneous tumor‐bearing BALB/c nude mice (*n* = 3 for each group). As shown in Figure 4g and Figure S20a (Supporting Information), the tumors could be clearly delineated with strong fluorescence even at 144 h after MPDA/ICG@M‐gy1 injection, indicating its considerable tumor specificity. In contrast, for MPDA/ICG@M, the signal was barely visible at the tumor site. The excellent tumor recognition capability of MPDA/ICG@M‐gy1 could also be confirmed through ex vivo imaging of the tumors and major organs collected from the mice sacrificed 144 h after injection. Quantitative analysis revealed a significantly higher MPDA/ICG@M‐gy1 accumulation at the tumor site compared with that of MPDA/ICG@M (Figure S20b, Supporting Information), consistent with the in vitro results. Furthermore, MPDA/ICG@M‐gy1 or MPDA/ICG@M were intravenously injected into 22RV1 bone metastasis tumor‐bearing BALB/c nude mice (*n* = 3 for each group), and the results of imaging were consistent with above (Figure 4h; Figure S21a,b, Supporting Information).

PDA has strong optical absorption in the near infrared (NIR) region.^[^ ^38^ ^]^ Consequently, we evaluated the MNP‐mediated photothermal efficacy in vivo by irritating the tumor region with an NIR laser (808 nm) after the injections. The results showed that MPDA@M‐gy1 significantly induced higher photothermal heating temperature in the tumor sites than MPDA@M and PBS, which further confirmed the specific targeting ability of MPDA@M‐gy1 (Figure S22a,b, Supporting Information).

To assess the enrichment of the nanoparticles in the CRPC tumor tissue, we injected MPDA/FITC@M‐gy1 or MPDA/FITC@M intravenously into 22RV1 subcutaneous tumor‐bearing BALB/c nude mice and obtained the pathological slices of the tumor tissues. CD31‐labeled tumor vessels were identified through red fluorescence, and MPDA/FITC@M‐gy1 and MPDA/FITC@M were located through green fluorescence. The immunofluorescence analysis indicated that MPDA/FITC@M‐gy1 in the internal tumor hypovascular zone had a significantly higher fluorescence intensity than MP DA/FITC@M (Figure 4i; Figure S23, Supporting Information). This antibody‐antigen binding method not only possessed good targeting performance, but also increased the ability of drugs to be deeply penetrated and permeated at the tumor site.

Taken together, these results above indicated that M‐gy1 use has advantages of both the macrophage membrane and gy1, endowing our MNPs with superior PSMA‐binding affinity and CRPC tumor–targeting capability—a prerequisite for targeted tumor therapy.

### 2.5 In Vitro Anti‐CRPC Effects of MPDA/Fe/RSL3@M‐gy1

**Figure 5:**
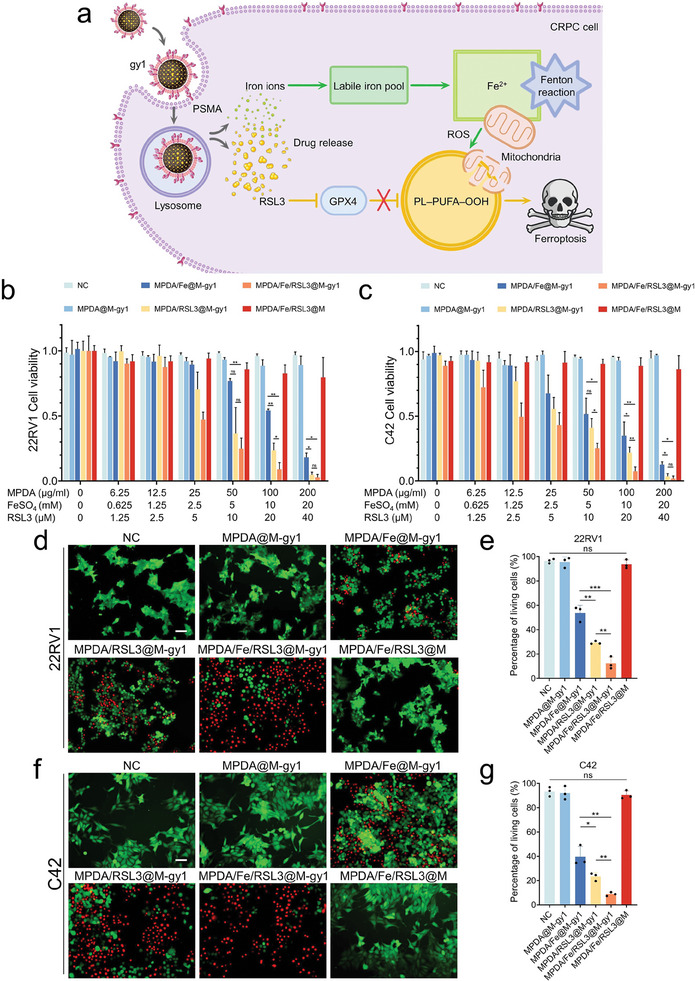
Anti‐CRPC effects of MPDA/Fe/RSL3@M‐gy1 in vitro. a) Schematic of CRPC‐targeted internalization of MNPs, and the mechanism underlying CRPC cell ferroptosis induction. b) Viability of 22RV1 cells treated with different concentrations of PBS, MPDA@M‐gy1, MPDA/Fe@M‐gy1, MPDA/RSL3@M‐gy1, MPDA/Fe/RSL3@M‐gy1, and MPDA/Fe/RSL3@M. c) Viability of C42 cells treated with different concentrations of PBS, MPDA@M‐gy1, MPDA/Fe@M‐gy1, MPDA/RSL3@M‐gy1, MPDA/Fe/RSL3@M‐gy1, and MPDA/Fe/RSL3@M. d) Live–dead cell imaging for surviving 22RV1 cells after incubation with different agents. Scale bar: 50 µm. e) Quantitative analysis of the percentage of living cells. f) Live–dead cell imaging for surviving C42 cells after incubation with different agents. Scale bar: 50 µm. g) Quantitative analysis of the percentage of living cells. Data are presented as means ± standard deviations from at least three independent experiments. ns, no significance. *p < 0.05, **p < 0.01, ***p < 0.001.

Next, we assessed the efficiency of MNPs in killing CRPC cells and the cell‐killing mechanism of the MPDA/Fe/MPDA@M‐gy1 is shown in **Figure** **5**a. We first evaluated the cytotoxicity of MPDA/Fe/RSL3@M‐gy1 toward CRPC cells. The results of CCK‐8 assay demonstrated that, compared with MPDA@M‐gy1 or MPDA/Fe/RSL3@M, MPDA/Fe/RSL3@M‐gy1, MPDA/Fe@M‐gy1 and MPDA/RSL3@M‐gy1 all displayed dose‐dependent toxicity toward 22RV1 and C42 cells, of which the half maximal inhibitory concentration (IC50) of MPDA/Fe/RSL3@M‐gy1 was the lowest (Figure 5b,c). In the 22RV1 and C42 cells, the IC50 of MPDA/Fe@M‐gy1 was 8.951 and 5.559 mm for FeSO_4_, respectively; that of MPDA/RSL3@M‐gy1 was 8.525 and 6.702 µm for RSL3, respectively; and that of MPDA/Fe/RSL3@M‐gy1 was 2.643 and 1.517 mм for FeSO_4_, respectively, and 5.285 and 3.034 µm for RSL3. Therefore, the MPDA/Fe/RSL3@M‐gy1 had the strongest cytotoxic effect (Figure S24a,b, Supporting Information). MPDA/Fe/RSL3@M‐gy1 had most significant cytotoxic effects in 22RV1 and C42 cells when FeSO_4_, RSL3, and MPDA doses were set at 10 mм, 20 µm, and 100 µg mL^−1^, respectively; (Figure 5b,c). MPDA/Fe/RSL3@M‐gy1 had the most significant cytotoxic effect, whereas MPDA/Fe/RSL3@M did not, confirming the highly targeted anti‐CRPC effect of MPDA/Fe/RSL3@M‐gy1. For further visual observations of MPDA/Fe/RSL3@M‐gy1 cytotoxicity, calcein‐AM and propidium iodide (PI) were used to costain live cells (green fluorescence) and dead cells (red fluorescence), respectively. The superior antitumor efficacy of MNPs with TM‐expressed gy1 against CRPC cells (22RV1 and C42) was further supported by the live–dead cell imaging results, in which the MPDA/Fe/RSL3@M‐gy1 led to the highest number of dead cells (Figure 5d–g). We further co‐incubated excess gy1 with 22V1 cells and then added the drug loaded nanoparticle MPDA/Fe/RSL3@M‐gy1. To further visualize MPDA/Fe/RSL3@M‐gy1 cytotoxicity, we co‐stained live cells and dead cells with calcein‐AM and PI, respectively. The live–dead cell imaging results showed that the MPDA/Fe/RSL3@M‐gy1 group had the highest number of dead cells compared to the blank control group and gy1+MPDA/Fe/RSL3@M‐gy1 group. This confirms the superior specific targeting efficacy of MNPs with TM‐expressed gy1 against CRPC cells (Figure S25a,b, Supporting Information).

### 2.6 In Vitro Ferroptosis Enhancement of MPDA/Fe/RSL3@M‐gy1

**Figure 6:**
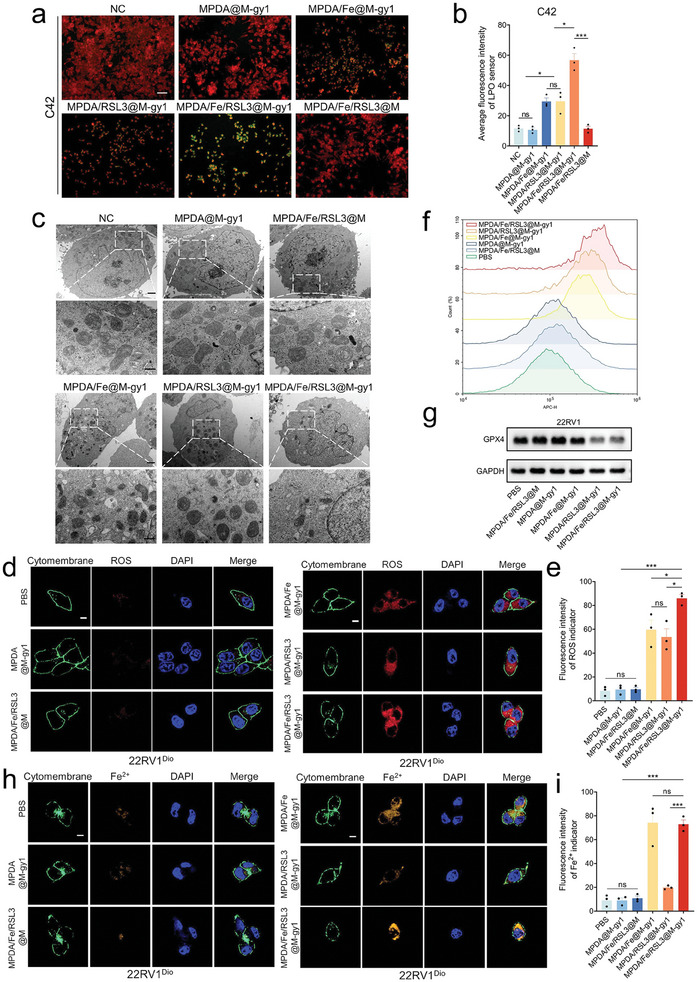
Ferroptosis enhancement of MPDA/Fe/RSL3@M‐gy1 in vitro. a) CLSM monitoring of intracellular lipoperoxide accumulation on 22RV1 cells after incubation with different agents. Scale bar: 50 µm. b) Quantitative analysis of the average fluorescence intensity using the LPO sensor in C42 cells. c) TEM for morphological changes in tumor mitochondria after incubation with different agents. d) CLSM monitoring of intracellular ROS accumulation on 22RV1 cells after incubation with different agents. Scale bar: 10 µm. e) Quantitative analysis of average fluorescence intensity of the ROS indicator in 22RV1 cells. f) FCM analysis of ROS levels in different agent–treated 22RV1 cells by using a ROS fluorescent probe (CellROX Deep Red probe). g) Western blots for intracellular expression of GPX4 in 22RV1 cells after incubation with different agents. h) CLSM evaluation of Fe^2+^ localization in 22RV1 cells incubated with different agents. Scale bar: 10 µm. i) Quantitative analysis of average fluorescence intensity of the Fe^2+^ indicator in 22RV1 cells. Data are presented as means ± standard deviations from at least three independent experiments. ns, no significance. *p < 0.05, **p < 0.01, ***p < 0.001.

The mechanism underlying ferroptosis involves the production and accumulation of ROS, particularly the highly toxic hydroxyl radicals via iron‐based Fenton reaction.^[^ ^39^ ^]^ As a key regulator of ferroptosis, GPX4 uses reduced glutathione (GSH) to convert lipid hydroperoxides to lipid alcohols, thereby alleviating LPO and inhibiting ferroptosis.^[^ ^40^ ^]^ Therefore, we designed the PSMA‐targeted MNPs to promote the accumulation of LPO by inhibiting GPX4, while simultaneously producing ROS by loaded iron ions, doubly promoting ferroptosis. To detect whether cell killing is achieved through ferroptosis, we first investigated the changes in LPO levels using a lipid peroxidation sensor BODIPY 581/591 C11. As shown in **Figures** **6**a,b and S26a,b (Supporting Information), quantitative CLSM imaging demonstrated that the MPDA/Fe/RSL3@M‐gy1 efficiently promoted LPO accumulation in both C42 and 22RV1 cells. The amount of LPO in the MPDA@M‐gy1 and MPDA/Fe/RSL3@M groups remained at a negligibly low level similar to the control group. The MPDA/Fe@M‐gy1 and MPDA/RSL3@M‐gy1 groups demonstrated a modest increase compared with their non–iron ion‐ or non–RSL3‐containing counterparts. Moreover, FCM analysis also confirmed the results mentioned above (Figure S27a,b, Supporting Information). These data demonstrated that the synergistic reaction effect of the iron ions and RSL3 could significantly increase LPO levels. Since LPO was an important functional marker of ferroptosis, lipidomic analysis was performed to study the changes of lipids in 22RV1 cells after MNPs treatment, using liquid chromatography‐mass spectrometry. We first assessed the levels of medium and long chain PUFAs substrates for peroxidation in 22RV1 cells. As Figure S28a (Supporting Information) showed that, treatment with the MPDA/Fe/RSL3@M‐gy1 group displayed the lowest levels of seven PUFAs substrates (C18:3N3, C18:3N6, C20:3, C20:4, C20:5, C22:4 and C22:5N6) in 22RV1 cells compared to other groups, suggesting its enhanced ability to promote PUFA oxidation and induce ferroptosis in 22RV1 cells. As the accumulation of PUFA‐phospholipids (PL‐PUFAs) such as phosphatidylcholine (PC), phosphatidylethanolamine (PE), phosphatidylglycerols (PG), phosphatidylserine (PS) and phosphatidylinositol (PI) plays an essential role in driving ferroptosis,^[^ ^41^ ^]^ we further assessed the levels of these 5 types of PL‐PUFAs (a total of 720) in 22RV1 cells. The result showed that compared to the control group, MPDA/Fe/RSL3@M‐gy1 group significantly increased the levels of 381 PL‐PUFAs, including PC (18:1e_19:1), PC (18:1e_20:3), PE (18:1_20:2), PE (20:1e_20:1), PG (19:1_18:1), PG (18:1_22:6), PI (17:0_20:4), PI (18:0_20:4), PS (18:1_18:1) and PS (18:1_20:4), which indicated that MPDA/Fe/RSL3@M‐gy1 could deplete PUFAs substrates and promote the PL‐PUFAs levels in 22RV1 cells, finally inducing LPO accumulation and leading to ferroptosis (Figure S28b, Supporting Information). The result above is consistent with the findings of Chuying Huang et al.^[^ ^42^ ^]^

In order to further explore the ferroptosis‐related killing mechanism of membrane‐coated nanoparticles on CRPC cells, we have performed an additional experiment of glutathione (GSH)/oxidized glutathione (GSSG) ratio in vitro using the GSH and GSSG Assay Kit. As Figure S29 (Supporting Information) showed that, the GSH/GSSG ratio was the lowest in the MPDA/Fe/RSL3@M‐gy1 group, followed by the MPDA/Fe@M‐gy1 group and the MPDA/RSL3@M‐gy1 group which were significantly lower than those in the other three groups. When iron ions enter the cell, the Fenton reaction is induced, significantly increasing the levels of free radicals and ROS, which can react directly with GSH, causing it to be consumed in large quantities.^[^ ^43^ ^]^ Simultaneously, although GPX4 is inhibited by RSL3, preventing GSH from rapidly catalyzing GSSG, this inhibition leads to the accumulation of hydrogen peroxide (H_2_O_2_) and lipid peroxide (Lipid‐OOH). As a result, the cells remain in a continuous state of oxidative stress, eventually causing a significant decrease in GSH levels.^[^ ^44^ ^]^ This result is consistent with the findings of Yuanyuan Shi et al.^[^ ^45^ ^]^ Additionally, as mitochondrial damage is a hallmark of ferroptosis, we observed the morphological and mitochondrial membrane potential (MMP) changes of mitochondria. As shown in Figure 6c, the amount of mitochondrial membrane in the MPDA/Fe/RSL3@M‐gy1 group decreased considerably, accompanied by decreases in the mitochondrial volume and increases in mitochondrial membrane density—a characteristic of ferroptosis‐induced mitochondrial dysfunction.^[^ ^46^ ^]^ We then performed an additional experiment by using the JC‐1 probe to detect the changes in MMP. When there is damage to the mitochondrial, the MMP level decreases, and the JC‐1 probe shifts from a multimeric state in the mitochondrial matrix (JC‐1 aggregate), resulting in increased green fluorescence (JC‐1 monomer). The FCM analysis shown in Figure S30a,b (Supporting Information) indicated that green fluorescence increased significantly in 22RV1 cells treated with the MPDA/Fe/RSL3@M‐gy1, compared to other groups. The results demonstrated that 22RV1 cells treated with the MPDA/Fe/RSL3@M‐gy1 exhibited the most significant mitochondrial damage due to ferroptosis.

Furthermore, the ROS levels in 22RV1 cells were assessed using CLSM imaging and FCM analysis with CellROX Deep Red (an ROS indicator). The MPDA/Fe/RSL3@M‐gy1 group showed the highest cellular ROS level; it was 9.2‐fold higher than that in the control group. The cellular ROS levels in the MPDA/Fe@M‐gy1 and MPDA/RSL3@M‐gy1 groups also demonstrated an increase compared with the MPDA@M‐gy1, MPDA/Fe/RSL3@M, and control group (Figure 6d–f; Figure S31, Supporting Information). Similarly, our western blot results revealed that GPX4 expression was significantly downregulated in the MPDA/Fe/RSL3@M‐gy1 group, validating its potent GPX4‐inhibiting capabilities in CRPC cells (Figure 6g). From a biochemical perspective, increasing the intracellular Fe^2+^ levels could increase the labile iron pool, which can catalyze the reaction between H_2_O_2_ and PUFAs on the cytoplasmic membrane, eventually leading to ferroptosis.^[^ ^47^ ^]^ The influence of MPDA/Fe/RSL3@M‐gy1 on the Fe^2+^ levels in 22RV1 cells was estimated using FerroOrange (a Fe^2+^ indicator). Our CLSM results demonstrated that the cellular Fe^2+^ levels were significantly higher in the MPDA/Fe@M‐gy1 and MPDA/Fe/RSL3@M‐gy1 groups than in the other groups, which demonstrated that the loaded iron ions, specifically taken up by the 22RV1 cells, participated in the Fenton reaction (Figure 6h,i).

Taken together, these results demonstrated that MPDA/Fe/RSL3@M‐gy1 could specifically kill CRPC cells by inducing ferroptosis.

### 2.7 In Vivo Anti‐CRPC Effects of MPDA/Fe/RSL3@M‐gy1

#### 2.7.1 Subcutaneous Tumor Model

**Figure 7:**
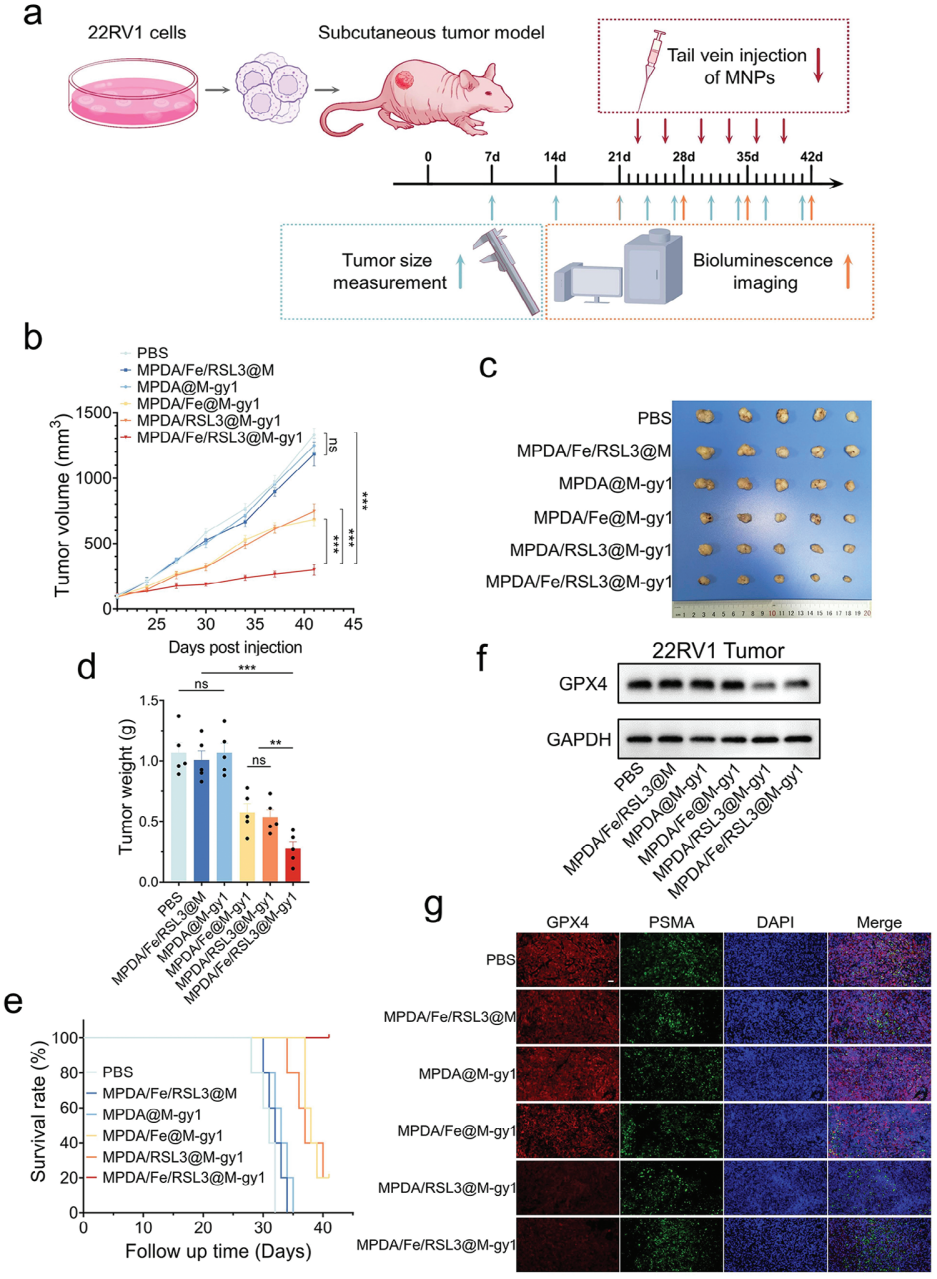
Anti‐CRPC effects of MPDA/Fe/RSL3@M‐gy1 in the subcutaneous tumor model. a) Schematic of dosing regimens and tumor growth monitoring in 22RV1 tumor‐bearing mice. b) Changes in tumor volumes in 22RV1 tumor‐bearing mice after different treatments (*n* = 5). c) Visual comparison of whole tumors from 22RV1 tumor‐bearing mice after treatment in different groups. d) Final weights of tumor tissues after treatment in different groups. e) Survival rate of 22RV1 tumor‐bearing mice in 42 days. f) Western blot analysis for protein expression levels in 22RV1‐tumor tissues of GPX4 after different treatments. g) Immunofluorescence images of GPX4 expression in 22RV1 tumor tissues after treatment in different groups. Scale bar: 50 µm. Data are presented as means ± standard deviations from at least three independent experiments. ns, no significance. **p < 0.01, ***p < 0.001.

Subsequently, we detected the therapeutic efficacy in a CRPC xenograft mouse model. Tumor‐bearing BALB/c nude mice were randomly divided into six groups (*n* = 5 per group): PBS, MPDA/Fe/RSL3@M, MPDA@M‐gy1, MPDA/Fe@M‐gy1, MPDA/RSL3@M‐gy1, and MPDA/Fe/RSL3@M‐gy1 groups. Tumor growth was monitored using a Xenogen In Vivo Imaging System (IVIS) for the detection of bioluminescence imaging or by measuring the average tumor size. The nude mice were randomly grouped 21 days after tumor implantation and subsequently injected with different concentrations of MNPs via the tail vein twice a week (**Figure** **7**a). The results of tumor measurement (Figure 7b–d; Figure S32a,b, Supporting Information) indicated that tumors in the MPDA@M‐gy1 and MPDA/Fe/RSL3@M groups grew rapidly, with their sizes almost comparable to those in the PBS group, suggesting the nontoxicity of the MPDA‐based nanostructures and the nontargeting ability of MNPs without TM‐expressed gy1. Nevertheless, MPDA/Fe@M‐gy1, MPDA/RSL3@M‐gy1, and MPDA/Fe/RSL3@M‐gy1 treatment led to tumor growth inhibition to varying degrees. The MPDA/Fe/RSL3@M‐gy1 group demonstrated the highest tumor inhibition ability, whereas no significant difference was noted in the tumor inhibition abilities of MPDA/Fe@M‐gy1 and MPDA/RSL3@M‐gy1.

Because a tumor volume of 600 mm^3^ is the standard in survival analysis, a tumor volume of ≥600 mm^3^ was considered a positive event, and that of <600 mm^3^ a negative event. We noted no positive event in the MPDA/RSL3@M‐gy1 group after six treatments, and the time of the positive event in the MPDA/Fe@M‐gy1 and MPDA/RSL3@M‐gy1 groups lasted longer than that in the PBS, MPDA/Fe/RSL3@M, and MPDA@M‐gy1 groups (Figure 7e). Therefore, the MPDA/Fe/RSL3@M‐gy1 group, loaded with two drugs, demonstrated the highest inhibitory effect on CRPC tumors in vivo. The excellent anti‐CRPC efficacy of the MPDA/Fe/RSL3@M‐gy1 group was further supported by IHC staining for Ki67. Synergistic treatment with iron ions and RSL3 led to considerable improvements in the anti‐CRPC effects and strongly suppressed tumor cell proliferation—consistent with our in vivo results described above (Figure S33a,b, Supporting Information). The proposed therapeutic mechanism of MNP‐activated ferroptosis was also supported by our in vivo Western blotting results, where GPX4 expression was significantly downregulated in the MPDA/Fe/RSL3@M‐gy1 and MPDA/RSL3@M‐gy1 groups (Figure 7f). As shown in Figure 7g and Figure S34 (Supporting Information), the tumor cells in each treatment group were co‐stained for GPX4 and PSMA for immunofluorescence staining; the results confirmed that the tumor cells were positive for PSMA. Further analysis confirmed that the fluorescence intensity for GPX4 in tumor tissues treated with PBS, MPDA/Fe/RSL3@M, MPDA@M‐gy1, and MPDA/Fe@M‐gy1 was high, but without any significant between‐group differences. Compared with the PBS, MPDA/Fe/RSL3@M, MPDA@M‐gy1, and MPDA/Fe@M‐gy1 groups, the fluorescence intensity of GPX4 in MPDA/RSL3@M‐gy1 and MPDA/Fe/RSL3@M‐gy1 treated groups was significantly lower, but without any significant between‐group differences; this result was consistent with that of Western blotting. Our analysis of terminal deoxynucleotidyl transferase (TdT)‐mediated dUTP nick end labeling (TUNEL) staining in post‐treatment tumor histology supports the treatment outcome and response rates. TUNEL staining results, in particular, showed the highest percentage of dead cells in the MPDA/Fe/RSL3@M‐gy1 group (Figure S35a,b, Supporting Information). This suggested that the MNPs‐mediated ferroptosis treatment could efficiently induce widespread tumor cell death in vivo.

We have performed additional experiments to assess LPO and ROS accumulation in vivo. Briefly, 22RV1 subcutaneous tumor‐bearing BALB/c nude mice were randomly divided into six groups (*n* = 5 per group) 15 days after tumor implantation and subsequently injected with different groups of MNPs (PBS, MPDA/Fe/RSL3@M, MPDA@M‐gy1, MPDA/Fe@M‐gy1, MPDA/RSL3@M‐gy1, and MPDA/Fe/RSL3@M‐gy1) via the tail vein twice a week. After a total of 8 treatments over 4 weeks, 22RV1 tumor tissues were harvested and isolated into primary tumor cells. All primary cells of 22RV1 tumor tissues were used for the subsequent studies. Primary cells of 22RV1 tumor tissues of each group were first co‐incubated with lipid peroxidation sensor BODIPY 581/591 C11 and the ROS indicator (CellROX Green), respectively. The results of FCM analysis indicated that the MPDA/Fe/RSL3@M‐gy1 group showed the highest LPO and ROS accumulation. (Figures S36a,b and S37a,b, Supporting Information).

In order to verify the tumor inhibition effect of the targeted induced ferroptosis strategy in immunocompetent mice, we have previously constructed a MyC‐CaP cell line stably expressing PSMA (MyC‐CaP^psma+^) and inoculated MyC‐CaP^psma+^ cells subcutaneously in immunocompetent FVB mice. The FVB mice were randomly grouped 14 days after tumor implantation and subsequently injected with different concentrations of MNPs via the tail vein twice a week. The result showed that MPDA/Fe/RSL3@M‐gy1 group demonstrated the highest tumor inhibition ability, which was consistent with tumor suppression in immunodeficient mice (Figure S38a–c, Supporting Information).

#### 2.7.2 Bone Metastasis Model

**Figure 8:**
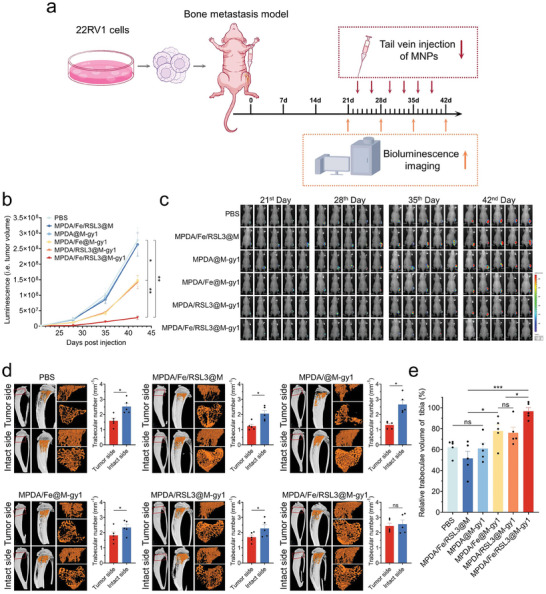
Anti‐CRPC effects of MPDA/Fe/RSL3@M‐gy1 in our bone metastasis model in different groups (n = 5). a) Schematic of dosing regimens and tumor growth monitoring in 22RV1 tumor‐bearing mice. b) Quantification of bioluminescence intensity. c) In vivo bioluminescence images of bone metastasis. d) Representative micro‐CT images of tibial metastases. e) Quantitative analysis of relative trabeculae volume of tibia. Data are presented as means ± standard deviations from at least three independent experiments. ns, no significance. *p < 0.05, **p < 0.01, ***p < 0.001.

PCa frequently metastasizes to bone where, in the CRPC setting, almost 90% of patients have detectable skeletal involvement.^[^ ^48^ ^]^ To date, bone metastases represent an incurable form of PCa and contribute significantly to disease‐specific mortality.^[^ ^49^ ^]^ To assess therapeutic potential of the active targeting MNPs‐mediated ferroptosis strategy to bone metastases, we established the CRPC orthotopic bone metastasis xenografts model, and administered treatments as described in **Figure** **8**a. Bioluminescence monitoring showed that the MPDA/Fe/RSL3@M‐gy1 group displayed the weakest bioluminescence intensity and resulted in optimal therapeutic outcomes (Figure 8b,c; Figure S39, Supporting Information). Furthermore, as illustrated by our micro‐computed tomography (micro‐CT) results, tumor‐induced tibial destruction, labeled using trabecular numbers, could reflect metastatic tumor growth. After treatment termination, the tumor‐ and intact‐side trabecular numbers did not show significant differences only in the MPDA/Fe/RSL3@M‐gy1 group; in the other groups, the tumor‐side trabecular numbers were significantly lower than those in the intact side (Figure 8d). Next, we calculated and analyzed the relative trabecular volume of the tibia (i.e., the ratio of tumor‐to‐intact–side trabecular numbers) in each group (Figure 8e). The results demonstrated that the MPDA/Fe/RSL3@M‐gy1 group had the highest relative trabeculae volume of the tibia, indicating that synergistic treatment demonstrated the most optimal anti‐CRPC efficacy.

In addition, we comprehensively evaluated MNPs’ neurotoxicity, peripheral, and organ toxicities. The body weight in the MPDA/Fe/RSL3@M‐gy1 group demonstrated no significant decrease compared with the control groups (PBS and MPDA@M‐gy1; Figure S27, Supporting Information). As shown in Figures S28a–f, S29, and S30 (Supporting Information), no obvious physiological abnormalities or systemic toxicity were observed in any of the treated mice, demonstrating that MPDA/Fe/RSL3@M‐gy1 may be safely applied as a reliable CRPC therapy platform.

## 3 Conclusion

In summary, our study confirmed the presence of high GPX4 expression in CRPC; on the basis of this result, we completed the effective CRPC‐targeting treatment by applying the GPX4 inhibitor RSL3 in combination with loaded iron ions. We designed an innovatively therapeutic nanoplatform based on engineered PSMA‐targeting macrophage membranes, encapsulating an MPDA core with ferroptosis‐inducing drugs for effective CRPC treatment. We proposed a robust strategy—to replace the conventional postfunctionalization approaches—where a PSMA‐specific gy1 is directly expressed on the macrophage membranes, which endows the MNPs with significantly increased circulation time and cell‐specific tumor targeting and internalization. The results demonstrated that our nanoplatform has the following advantages:

1. We, for the first time, achieved CRPC treatment through the induction of synergistic action of the GPX4 ferroptosis defense mechanism and MNP‐mediated iron ion release.
2. Taking advantage of the aromatic rings and catechol groups in dopamine, our MPDA‐based nanoplatform has high iron ion and RSL3 EE based on π–π stacking and mesoporous structure, and it can release iron ion and RSL3 in a responsive manner under acid response stimulation of lysosomal pathway.
3. Our conjugated nano‐system can achieve local targeting of CRPC tumors through a PSMA‐specific gy1 on the macrophage membrane.
4. The engineered MNPs have good biocompatibility and safety; consequently, synergistic CRPC treatment considerably inhibits tumor growth, micrometastasis, and concomitant damage.

Taken together, our results demonstrated that MPDA/Fe/RSL3@M‐gy1 can become a versatile platform for enhancing targeted nanomedicine delivery to tumor sites and improving the relevant therapeutic outcomes. Our study may provide a novel CRPC treatment strategy and broaden the prospects for precise treatment of CRPC in the future.

## 4 Experimental Section

### Materials

Dopamine hydrochloride (D103111), pluronic F‐127 (F127; P434421), 1,3,5‐trimethylbenzene (≥98%; T139982), ammonia solution (25%–28%; A112077), ICG (I423619), FeSO_4_·7H_2_O (F116338), and Rho (R104960) were purchased from Aladdin. RSL3 (HY‐100218A) was procured from MedChemExpress. 4′,6‐diamidino‐2‐phenylindole (DAPI; P0131), cell lysis buffer for Western and IP (P0013), horseradish peroxidase (HRP)‐labeled goat antirabbit IgG (A0208), cell plasma membrane staining kit with Dio and Dil (C1993 and C1991, respectively), and calcein–PI cell viability/cytotoxicity assay kits (C2015) were obtained from Beyotime Biotechnology. Anti‐PSMA (ab76104), anti‐GPX4 (ab125066), Alexa Flour 488 (ab150157; ab150113), and Alexa Flour 594 (ab150080) were procured from Abcam. Anti‐GAPDH (10494‐1‐AP) was purchased from Proteintech. TRIzol (15 596 026) and CellROX Deep Red (C10491) were obtained from Thermos Fisher Scientific. HiScript 1st strand cDNA synthesis kit (R111‐01) was procured from Vazyme. qPCR direct TB green premix (638 319) was purchased from Takara. The cell culture media Dulbecco's modified Eagle's medium (DMEM) and Roswell Park Memorial Institute (RPMI) 1640, as well as fetal bovine serum (FBS), were obtained from Gibco. FeRhoNox‐1 (a Fe^2+^ indicator; MX4588) was purchased from Shanghai Maokang Biotechnology. 22RV1 cells were obtained from Procell, whereas C42, MyC‐CaP, and Raw264.7 cells were obtained from American Type Culture Collection.

### Patient Specimens

This study was approved by the Medical Ethics Committee of the First Affiliated Hospital of the Fourth Military Medical University (approval number: KY20203274‐1), and all participating patients provided their written informed consent. Tissue samples were obtained from patients who underwent radical prostatectomy or transrectal prostate puncture surgery for the detection of PSMA and GPX4 expression, followed by IHC staining

### IHC Staining

Tissue sections (thickness, 4 µm) were subjected to antigen retrieval in a citrate solution. After they were blocked with goat serum for 30 min at room temperature, the sections were incubated with primary antibodies against PSMA and GPX4 in a humidified box at 4 °C overnight. This was followed by incubation with secondary antibodies conjugated with Alexa Fluor 488 and Alexa Fluor 594 for 2 h at room temperature. Nuclei were stained using DAPI. Digital images were acquired through CLSM (FV31s‐sw; Olympus). Quantitative analysis was performed using Image J.

### Human Single‐Cell Sequencing Analysis

Resource datasets were downloaded from the Gene Expression Omnibus (GEO) database. The count matrix was subsequently converted to the “dgCMatrix” format. The “RenameCells” method was used to ensure that all cell labels were unique. Furthermore, quality control was applied to the cells based on several criteria. In brief, cells with <200 detected genes or those with >20% mitochondrial content were removed. To ensure uniform gene expression across cells, the “LogNormalize” function was used with a scale factor of 10000. The top 2000 genes exhibiting expression variability were selected for further analysis by using the “FindVariableFeatures” method. To eliminate unwanted sources of variation, the “ScaleData” function was employed with the “vars.to.regress” option, considering unique molecular identified and percent mitochondrial content. The dimensionality of the dataset was reduced by incorporating highly variable features into principal component analysis, which facilitated the identification of the first 30 principal components for subsequent analysis. Clustering analysis was performed by considering the edge weights between cells, and a shared nearest‐neighbor graph was generated using the Louvain algorithm, which was implemented through the “FindNeighbors” and “FindClusters” functions. The UMAP method was employed to visualize the identified clusters. To conduct subclustering analysis, we used a similar procedure, encompassing normalization and selection of variably expressed features, reduction of dimensions, and identification of clusters.

### Data Availability

All the expression data can be obtained from the public databases. ScRNA‐seq data of patients with HSCP and CRPC are available in the GEO databases: GSE176031 and GSE137829.

### Cell Culture and Lentivirus Infection

The cell lines MyC‐CaP and Raw264.7 (murine macrophages) were cultured in DMEM, whereas the cell lines 22RV1 and C42 were cultured in RPMI 1640. Both the aforementioned media contained 10% FBS and 1% penicillin–streptomycin, and the culture conditions were 37 °C in humidified air with 5% CO_2_. Lentivirus used for Raw264.7 infection was synthesized based on the vector GV218 (GeneChem). Three days after lentivirus infection (multiplicity of infection = 30), M‐gy1 with stable gy1 TM expression was screened for with 3 µg mL^−1^ puromycin. Then, M‐gy1 was harvested, washed, and resuspended in fluorescence‐activated cell sorting (FACS) buffer at 1 × 10^6^ cells mL^−1^ for FCM (Beckman Coulter) analysis.

### Animal Models

Male BALB/c nude mice and FVB mice (age, 6–8 weeks) were purchased from the Animal Experimental Center of the Fourth Military Medical University. All animal experiments were approved by the Institutional Animal Experiment Administration Committee of the First Affiliated Hospital of the Fourth Military Medical University. All animals were fed in specific pathogen‐free conditions. All surgical procedures were performed under anesthesia. Xenograft volumes [= long axis × (short axis)^2^/2] were measured weekly. Simultaneously, an IVIS imaging system (Xenogen) was used to monitor the volume

To obtain the castration‐resistant subcutaneous‐cell–derived xenograft model, 8 × 10^6^ MyC‐CaP cells mixed with Matrigel (1:1) were injected into the flanks of male FVB mice. To obtain the PDX model, patient tissues were trimmed into 1 ×1 × 1‐mm^3^ pieces, and then mixed with Matrigel (1:1) were injected into the flanks of male BALB/c nude mice. When the volume of xenograft reached 200 mm^3^, mice were randomized into two groups: HSPC and CRPC. CRPC group mice were castrated through surgery, whereas HSPC group mice received a sham operation. After surgery, the CRPC group xenograft volume became smaller gradually but then grew rapidly. After the volume reached 800 mm^3^, mice from both groups were euthanized, and their xenografts were isolated for further studies.

For in vivo experiments, 1 × 10^7^ 22RV1 cells mixed with Matrigel (1:1) were injected into the flanks of male BALB/c nude mice. Moreover, 1 × 10^6^ 22RV1 cells were injected into the marrow cavity of the tibia of male BALB/c nude mice. ICG was loaded to detect the distribution of the nanoparticles in vivo. MPDA/ICG@M‐gy1 and MPDA/ICG@M were injected through the tail vein; this was followed by monitoring of the fluorescence intensity on the IVIS imaging system. All mice were sacrificed at the last monitoring point. Major organs were collected for further imaging, hematoxylin and eosin (H&E) staining, and CLSM.

### Western Blot Analysis

To obtain protein samples, cells and tissues were lysed in RIPA buffer. Equal amounts of protein samples were separated through 15% sodium dodecyl sulfate polyacrylamide gel electrophoresis and then transferred into polyvinylidene fluoride membranes. After they were blocked, the membranes were then incubated with appropriate primary antibodies and HRP‐conjugated secondary antibodies. The blots were visualized by using an electrochemiluminescence system.

### qPCR Analysis

Total RNA was extracted from the isolated tissues by using TRIzol, according to the manufacturer's protocol. cDNA synthesis was performed using the first‐strand cDNA synthesis kit. The relative gene expression was analyzed using TB green premix. The target mRNA expression was normalized to *GAPDH*. The primer sequences used here are listed in **Table** **1**.

**Table 1:** Primer sequences used in this study.

Gene	Primers (5′−3′)
*Mus musculus GPX4*	Forward: TGTGCATCCCGCGATGATT Reverse: CCCTGTACTTATCCAGGCAGA
Human *GPX4*	Forward: GAGGCAAGACCGAAGTAAACTAC Reverse: CCGAACTGGTTACACGGGAA
*M. musculus GAPDH*	Forward: AGGTCGGTGTGAACGGATTTG Reverse: GGGGTCGTTGATGGCAACA
Human *GAPDH*	Forward: GGAGCGAGATCCCTCCAAAAT GGCTGTTGTCATACTTCTCATGG

### Membrane Derivation

Plasma membranes of M‐gy1 were collected according to a previously published protocol.^[^ ^50^ ^]^ In brief, 1 × 10^7^ M‐gy1 cells were washed with PBS three times and resuspended in 1 mL of hypotonic lysing buffer, followed by ultrasonic fragmentation. The homogenized solution was centrifuged at 3500 g for 5 min at 4 °C, and the supernatant was centrifuged again at 16 900 g for 30 min at 4 °C. The precipitates were washed with DNase‐ and RNase‐free water and stored at −80 °C for subsequent analysis.

### MPDA Synthesis

MPDA with mesoporous structure was synthesized using a reported method,^[^ ^32^ ^]^ with slight modifications. A typical reaction mixture of 0.5 g of F127, 0.5 g of dopamine hydrochloride, and 0.8 mL of 1,3,5‐trimethylphenyl was dispersed in a mixture of 50 mL of water and 50 mL of ethanol through ultrasonication to form an emulsion solution. Then, 2.5 mL of ammonia was added to the reaction mixture with stirring at 40 °C. The product was collected through centrifugation after 4 h of reaction and washed with deionized water three times.

### MPDA/Fe/RSL3 Preparation

We mixed 1 mg of MPDA, 30 mg of FeSO_4_·7H_2_O, and 100 µg of RSL3 in 10 mL of deionized water to achieve the approximate drug concentration ratio (10 mm FeSO_4_, 20 µm RSL3, and 100 µg mL^−1^ MPDA). After 3 min of ultrasonication, the mixture was stirred at room temperature for 4 h in a dark, nitrogen‐free environment. The product was collected via centrifugation at 12 000 rpm for 10 min and washed with deionized water three times.

### MPDA/Fe/RSL3@M‐gy1 Preparation

Next, 200 µg of MPDA/Fe/RSL3 and 800 µg of M‐gy1 were mixed in 1 mL of deionized water. After 5 min of ultrasonication in an ice bath, the mixture was extruded using Avanti Polar Lipids with 400‐ and 200‐nm filter membranes (Whatman) successively. Membrane fusion was facilitated using a 10× continuous extrusion process. For in vivo treatment experiments, the collected nanoparticles were fully resuspended with 1 mL of PBS and injected 200 µL per mouse via the tail vein for each in vivo treatment.

### MNP Structure and Physical Properties

MNP structure and morphology were characterized through SEM and TEM. The size distribution and surface charges were measured using Zetasizer (Malvern). Iron ion and RSL3 loadings were analyzed on a UV‐vis spectrophotometer (Shimadzu). The compositional changes were analyzed through FTIR spectroscopy (Walthem). The valence state of the iron ions was analyzed through XPS (Thermo).

### Immune Evasion Assay

Rho was mixed with MPDA@M‐gy1 or MPDA at a stoichiometric ratio of 2:1. The reaction mixture was stirred at room temperature for 4 h for Rho loading to obtain MPDA/Rho@M‐gy1 and MPDA/Rho. MDPA/Rho@M‐gy1 and MPDA/Rho were incubated with Raw264.7 cells in an incubator for 24 h. After they were washed with PBS twice, the cells were fixed with 4% paraformaldehyde, visualized through CLSM, and quantified using Image Pro Plus.

### Cellular MNP Uptake Evaluation

To evaluate the PSMA‐targeting properties of the MNPs, we stained Raw264.7gy1 and Raw264.7 cells using the Dio staining kit, according to the manufacturer's protocol. The membranes were then harvested to prepare MPDA@M‐gy1 and MPDA@M. Next, 22RV1 and C42 cells were harvested, washed, and resuspended in an MNP suspension, followed by incubation at 4 °C for 30 min in the dark. After they were washed, the cells were analyzed through FCM.

CLSM was also used to detect the targeting ability. In brief, 1 × 10^4^ 22RV1 or C42 cells were seeded on a confocal dish and then stained using the Dil staining kit; next, the nanoparticles were coated with the Dio‐stained membranes to obtain MPDA@M‐gy1 or MPDA@M. The cells and MNPs were co‐incubated at 4 °C for 30 min. Then, they were washed with PBS twice and fixed with paraformaldehyde. Before visualization through CLSM, the cells were sealed with DAPI.

### Cell Viability

To evaluate cell viability, the cells were seeded into a 96‐well plate and a confocal dish and then treated with the indicated nanoparticles. Cells in the 96‐well plate were assessed using the CCK‐8 kit, according to the manufacturer's protocol. For cells in the confocal dish, calcein/PI cell viability assay was used for CLSM, according to the manufacturer's instructions. In brief, the MNPs were incubated with the cells in the confocal dish overnight; next, CLSM was performed to detect fluorescence in the cells.

### In Vitro ROS Detection

We cultured 1 × 10^4^ 22RV1 cells in a confocal dish for CLSM and 2 × 10^5^ 22RV1 cells in a six‐well dish for FCM. The nanoparticles were prepared and incubated with the cells overnight. Next, 2 µL of an ROS probe was added into the medium, followed by incubation at 37 °C for 30 min in the dark. Then, the cells were washed with PBS three times. The cells in the confocal dish were fixed with 4% paraformaldehyde and visualized through CLSM. The cells in the six‐well plates were collected and detected through FCM.

### Mitochondrial Morphology Evaluation

We seeded 4 × 10^5^ 22RV1 or C42 cells seeded into six‐well plates. The nanoparticles were prepared as mentioned above and incubated with the cells overnight. After they were washed with PBS three times, the cells were centrifuged at 800 rpm for 5 min. After 24 h of fixation with 2.5% glutaraldehyde, the cells were dehydrated using an alcohol gradient. Cell samples were then embedded, sectioned, stained, and observed through TEM.

### In Vitro Fe^2+^ Detection

In total, 1 × 10^4^ 22RV1 cells were seeded into a confocal dish and co‐incubated with the nanoparticles overnight. Next, the cells were washed with PBS and mixed with 2 µL of the Fe^2+^ indicator, followed by 30 min of incubation in the dark. Cells were then washed with PBS three times, fixed with 4% paraformaldehyde, and visualized through CLSM.

### Harvesting of Primary Tumor Cells

Tumor tissues were minced with scissors, then enzymatically digested in CO_2_‐independent incubator shaker (#ISF1‐XC, Kuhner, Germany) with 1 mg mL^−1^ Collagenase I (#SCR103, Sigma‐Aldrich, USA) and IV mixture (#C4‐28‐100MG, Sigma‐Aldrich, USA) for 1 h at 37 °C under 80 rpm agitation. After diluted with serum‐free medium and centrifugation at 200 g for 10 min, the cell pellets were resuspended in ACK lysis buffer (#NC9067514, ThermoFisher, USA) to remove blood cells. Prior to staining, the cell suspended in FACS buffer (PBS with 5% BSA, #SRE0096, Sigma‐Aldrich, USA) were filtered through a 40 µm mesh (#BS‐40‐XBS, Biosharp, China).

### Tibia Destruction Assessment

We injected 1 × 10^6^ 22RV1 cells into the marrow cavity of the tibia of male BALB/c nude mice. The mice were euthanized at treatment termination. Both tibias were excised, and the muscle tissue surrounding the bones was removed. The healthy‐side tibia was considered the control. The tibias were placed in the coronal plane, parallel to the detection tube of a micro‐CT platform (Skyscan 1276; Bruker). Images were reconstructed into 3D models by using NRecon and rendered using CTvox. Data Viewer was used for image visualization, and CTan was used to intercept and check the regions of interest for bone mass analysis.

### MNP General Toxicity Evaluation

Normal BALB/c nude mice were divided into three groups (n = 3) to receive tail‐vein injections of PBS, MPDA@M‐gy1, or MPDA/Fe/RSL3@M‐gy1 every 3 days. During the 2‐week observation period after each injection, the mouse body weights were monitored.

### MNP Organ Toxicity Evaluation

After 2 weeks of PBS, MPDA@M‐gy1, or MPDA/Fe/RSL3@M‐gy1 injection, the mice were sacrificed through neck dislocation, and blood samples were obtained from the left ventricle of the heart. Next, 200 µL of serum was obtained from the collected blood samples through centrifugation at 3000 rpm and 4 °C for 10 min. Serum biochemical markers including alanine aminotransferase (ALT), aspartate transaminase (AST), blood urea nitrogen (BUN), serum creatinine (CR), CR kinase (CK), and lactate dehydrogenase (LDH) were detected using commercial kits and a multifunctional biochemistry analyzer (AU600; Olympus), according to the manufacturers’ instructions. Moreover, the major organs—the brain, heart, lungs, liver, kidneys, intestines, and spleen—of mice injected with PBS, MPDA@M‐gy1, or MPDA/Fe/RSL3@M‐gy1 were resected and fixed with 4% paraformaldehyde. Fetal mice were collected from the pregnant mice 2 weeks after MNP injection. After they were embedded in paraffin, the tissues were sliced in 4‐µm‐thick sections and then subjected to H&E staining. The digital images of the stained sections were acquired using an inverted microscope (TE2000‐S; Nikon).

### Statistical Analysis

All data are expressed as means ± standard deviations. We compared two groups using Student's *t* test, whereas to compare multiple groups, we used one‐way analysis of variance. Each data was measured at least three times. All statistical analyses were performed on GraphPad Prism 8 and SPSS (version 27.0). A *p* value of <0.05 was considered to indicate significance.

## Conflict of Interest

The authors declare no conflict of interest.

## Supporting information

Supporting Information

## Data Availability

Data sharing is not applicable to this article as no new data were created or analyzed in this study.
