# Supplementary material for: Genetically Engineered Membrane‐Coated Nanoparticles for Enhanced Prostate‐Specific Membrane Antigen Targeting and Ferroptosis Treatment of Castration‐Resistant Prostate Cancer
Source: Adv Sci (Weinh). 2024 Jul 1;11(33):2401095. doi: 10.1002/advs.202401095 (PMC11434221; doi:10.1002/advs.202401095)
Supplement: Supplementary file 1 — Supporting Information [file ADVS-11-2401095-s001.docx]

**Supporting Information**


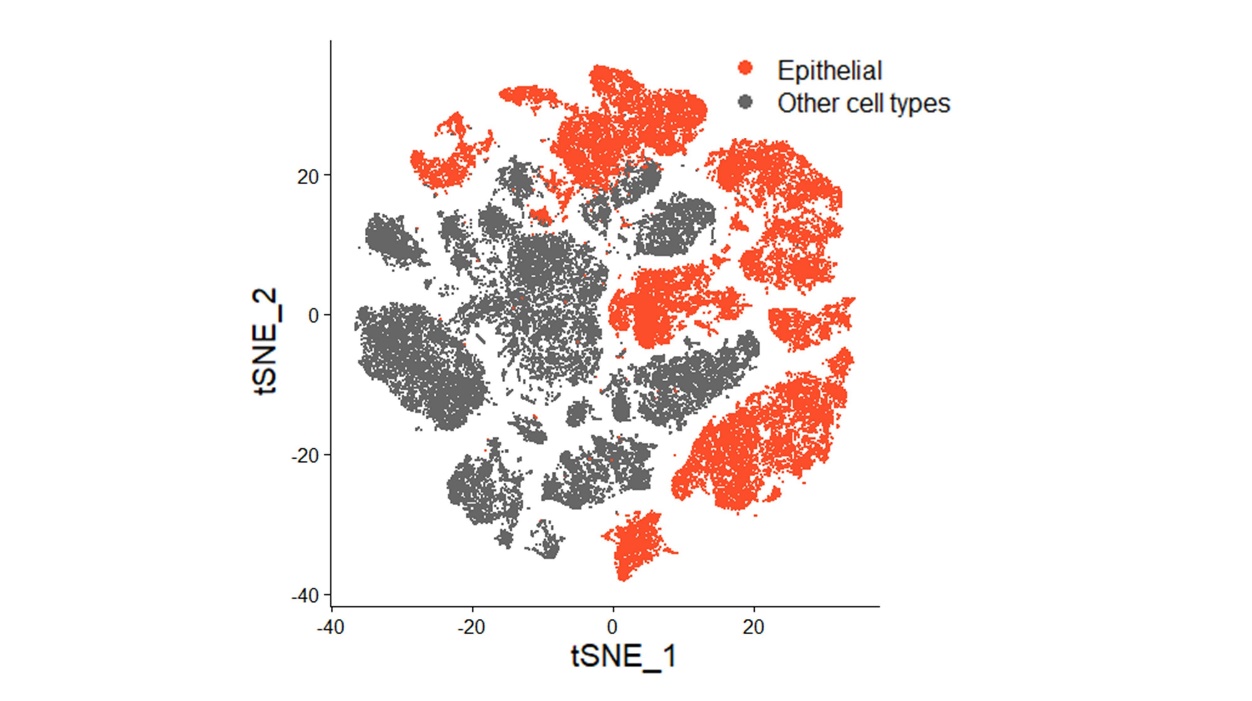


Figure S1 UMAP plot of the single cells profiled in the study, colored by epithelial cell types in prostate cancer.


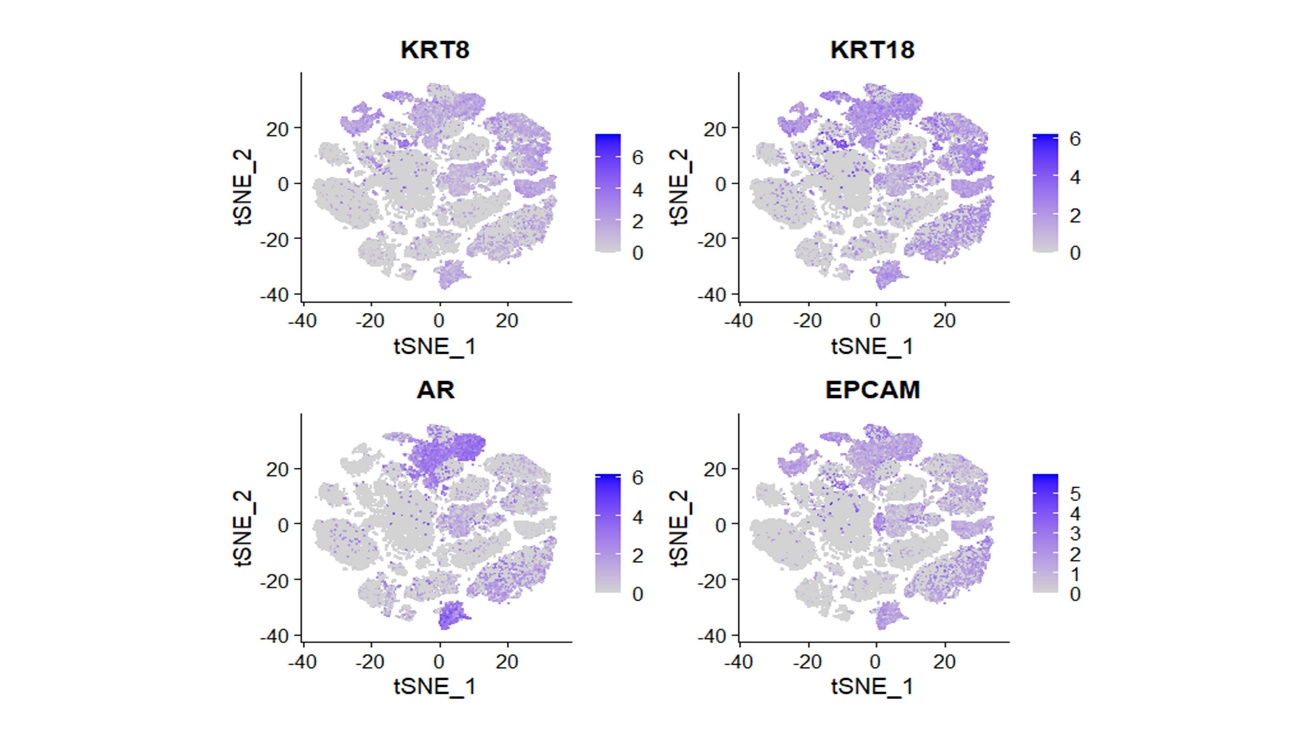


Figure S2 UMAP plot of the representative markers for epithelial cell.


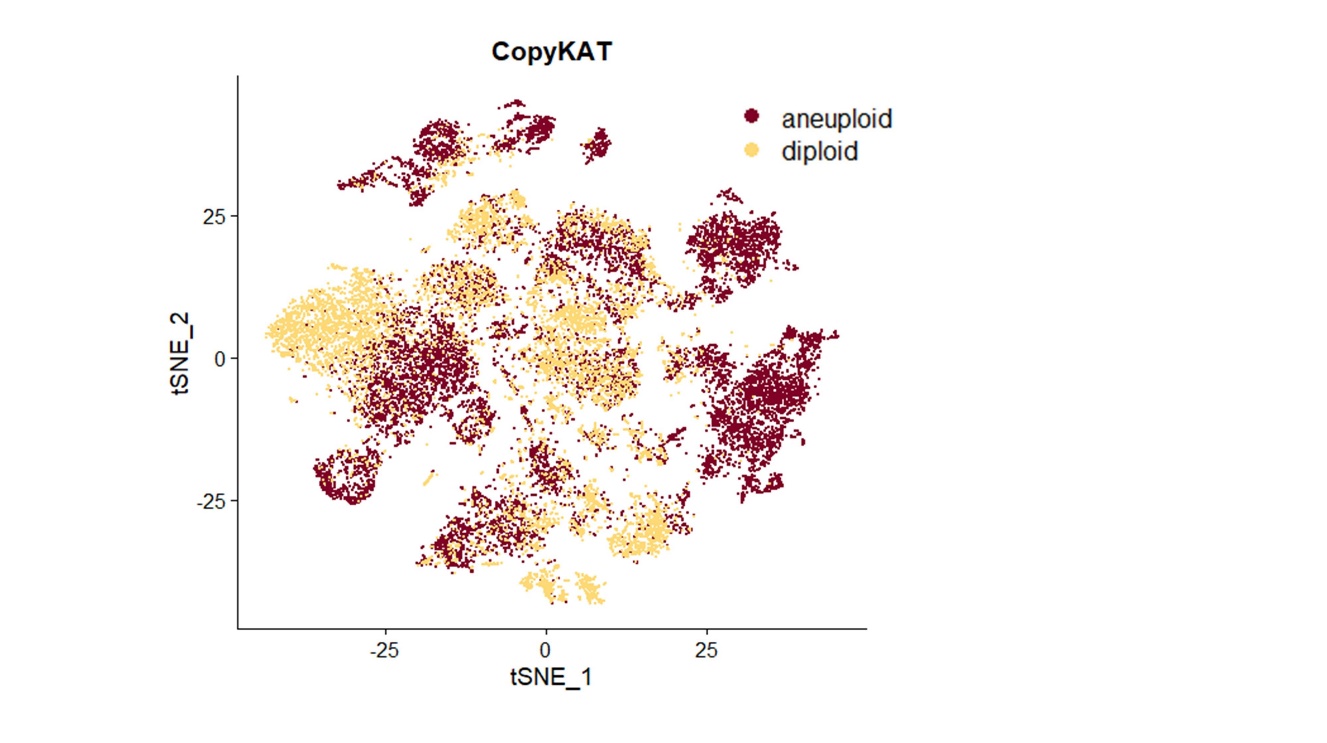


Figure S3 UMAP plot of the aneuploid and diploid cells in this integrated ScRNA-sequencing data.


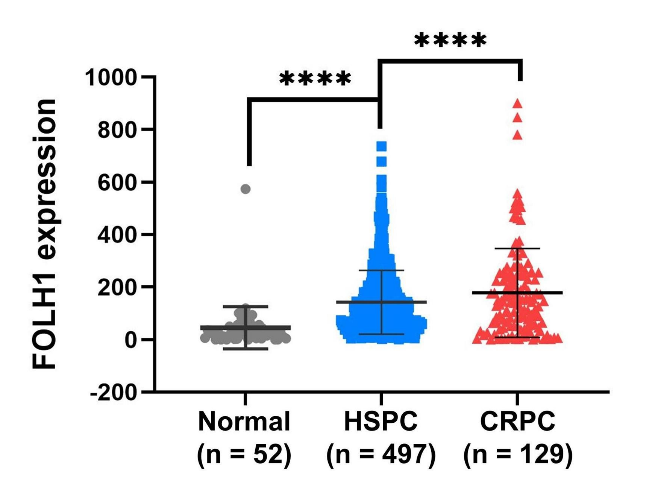


Figure S4 Comparison of *FOLH1* (PSMA) expression levels in prostate cancer adjacent tissues, HSPC tissues, and CRPC tissues in TCGA and cBioPortal database. ****p < 0.0001.


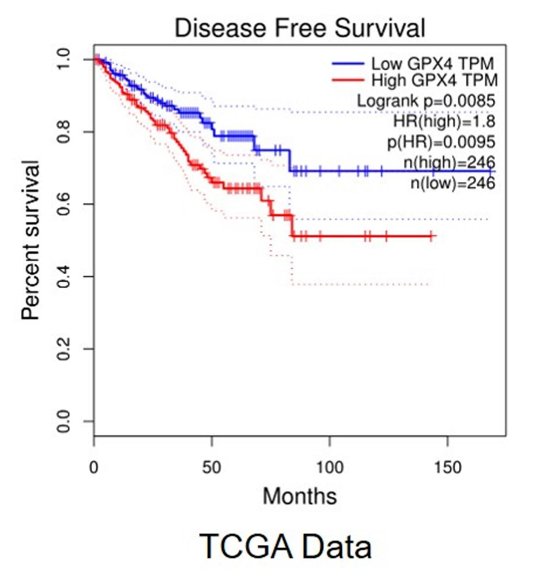


Figure S5 Disease free survival of PCa patients from the GEPIA database.


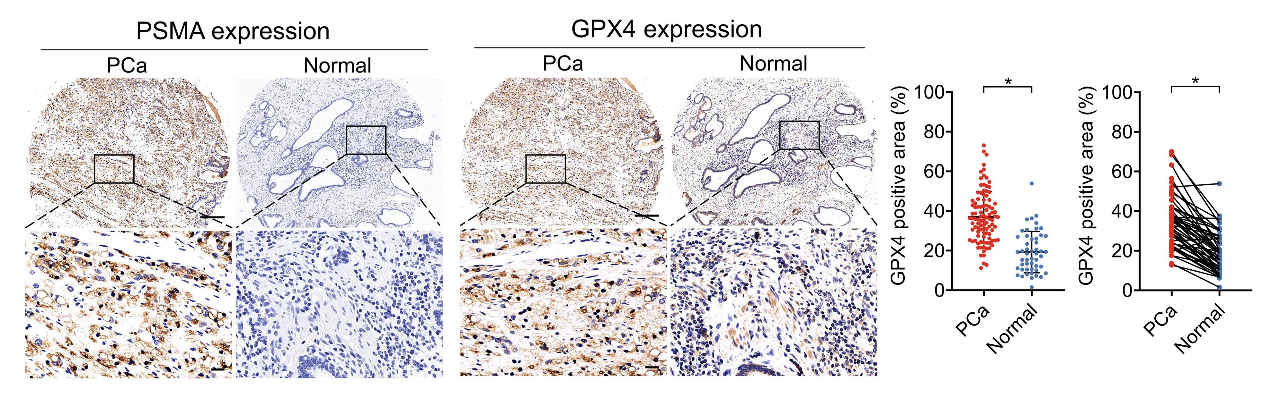


Figure S6 Representative IHC staining to show different GPX4 expression levels in PCa, using PSMA as the control. Scale bars: 200 μm and 50μm. Comparison and matched-Pair analysis between GPX4 expression levels in PCa and Normal tissue. The data are presented as the means ± SD. *p < 0.05.


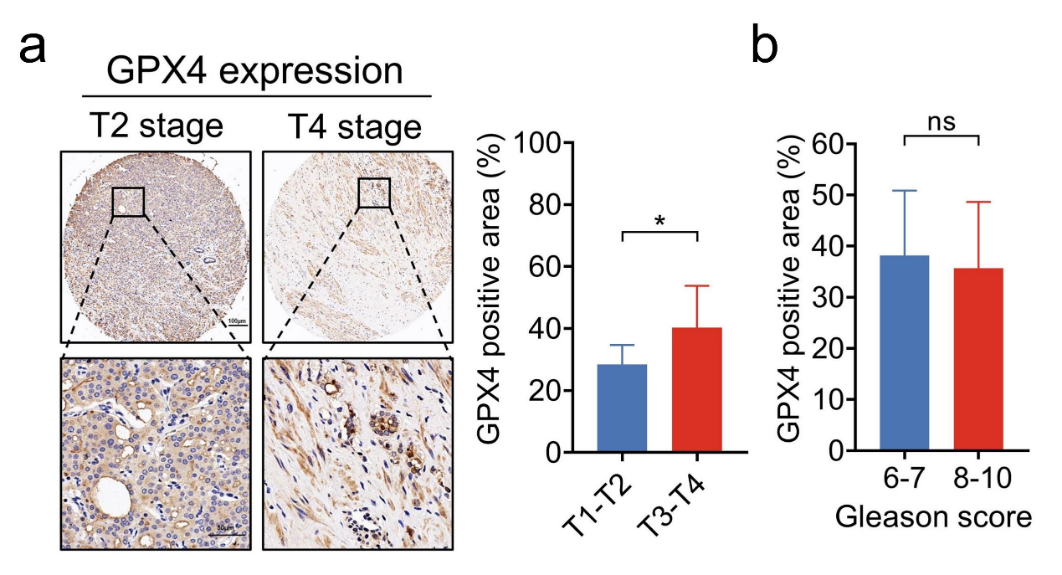


Figure S7 a) Representative IHC staining to show GPX4 expression levels in PCa tissues with different clinical stage. Scale bars: 200 μm and 50μm. b) Comparison between GPX4 expression and clinical stage. ns represented no significance, *p < 0.05.


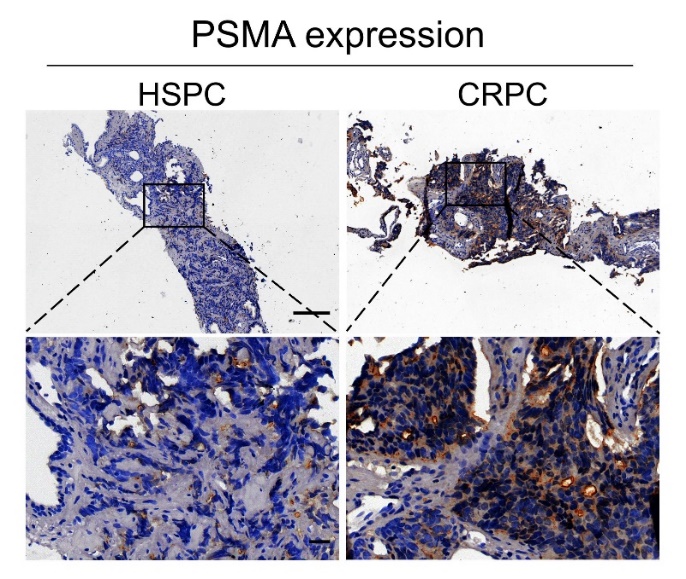


Figure S8 Representative IHC staining to show PSMA expression in PCa tumor tissues at HSPC and CRPC stages. Scale bars: 200 μm and 50μm.


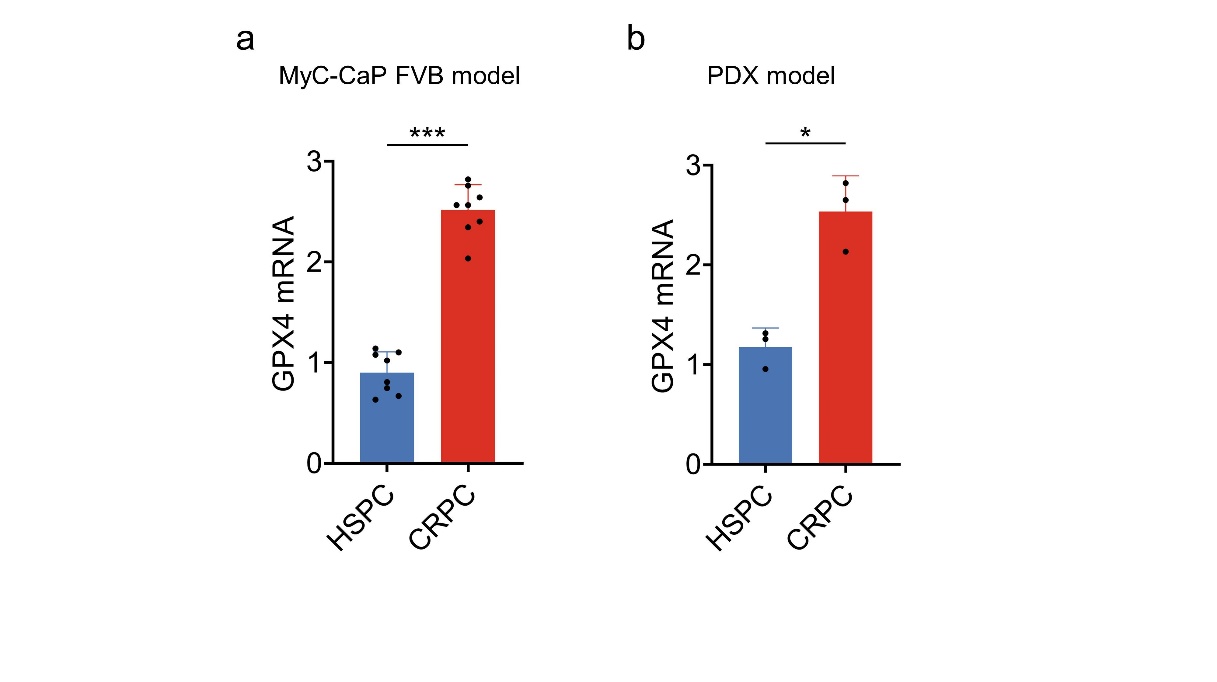


Figure S9 a) Comparison between GPX4 expression levels of HSPC and CRPC stage detected by qRT-PCR in different tumor-bearing models. a) MyC-Cap FVB model PDX model. b). *p < 0.05. ***p < 0.05.


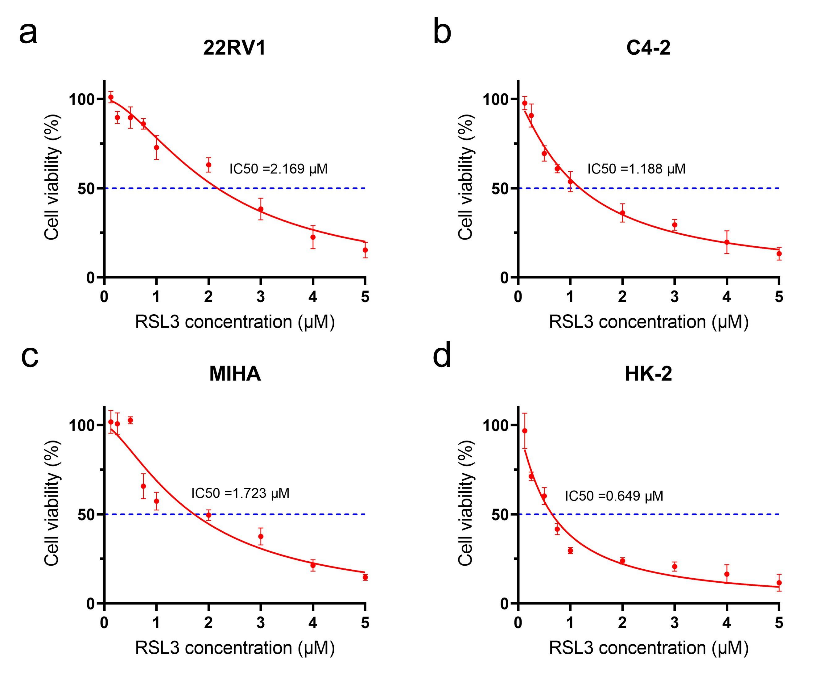


Figure S10 Cell viability and IC50 of different cell lines following RSL3 treatment for 72 hours. a) 22RV1 cell line. b) C42 cell line. c) MIHA cell line. d) HK-2 cell line.


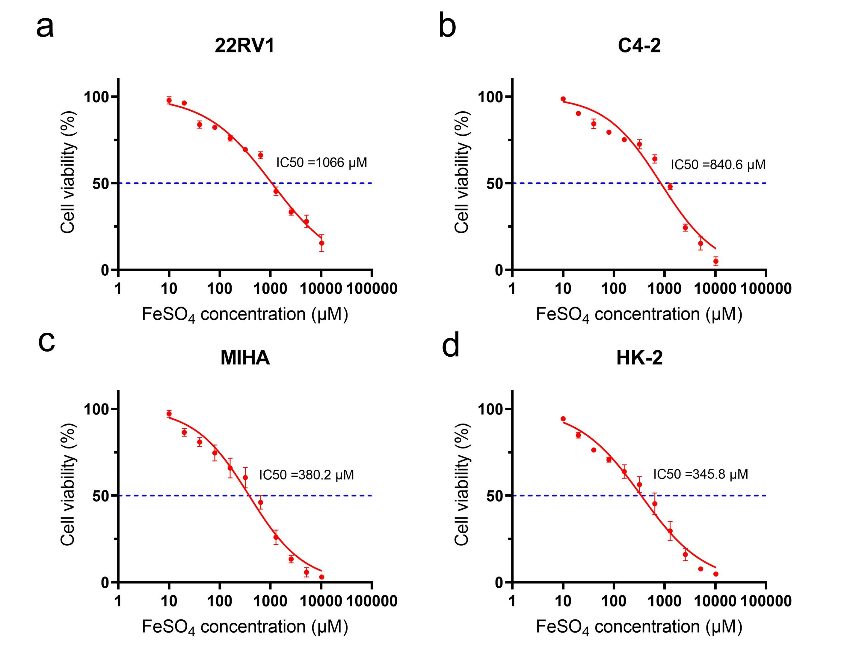


Figure S11 Cell viability and IC50 of different cell lines following FeSO_4_ treatment for 72 hours. a) 22RV1 cell line. b) C42 cell line. c) MIHA cell line. d) HK-2 cell line.


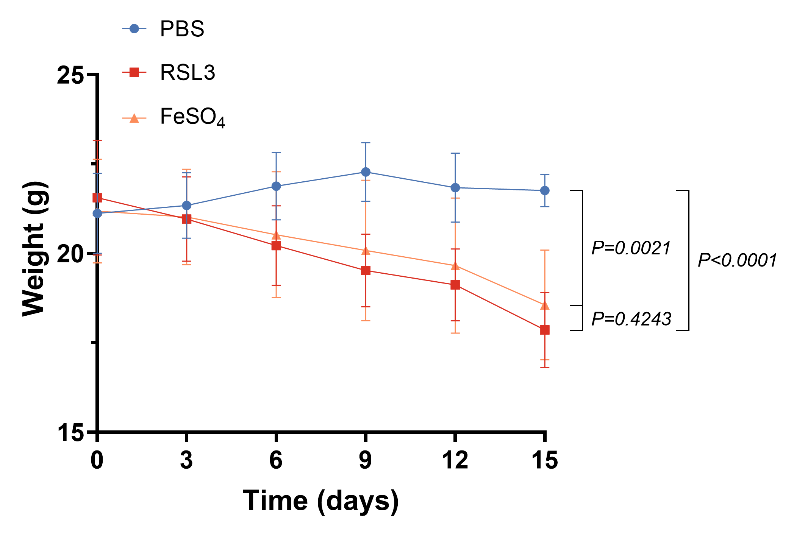


Figure S12 Quantitative comparison of mice weight of 22RV1 subcutaneous tumor-bearing BALB/c nude mice after the intravenous injection with PBS, RSL3 and FeSO_4_ for 15 days.


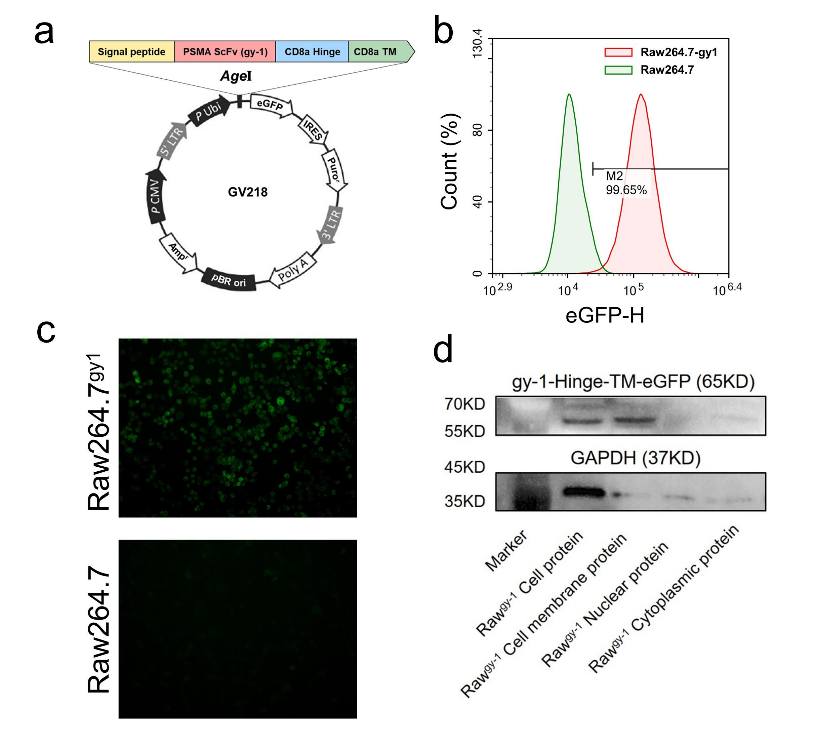


Figure S13 a) Schematic illustration of lentiviral vector used to construct M-gy1. b) gy1-eGFP fusion protein expression in M-gy1 detected using flow cytometry. c) Fluorescence imaging of puromycin-resistant macrophagegy-1 cells (M-gy1). Transmembrane (TM)-expressed eGFP was used as fluorescence label of M-gy1. d) Western blotting of fusion protein. The observed molecular weight of fusion protein was consistent with the predicted value 65 kDa.


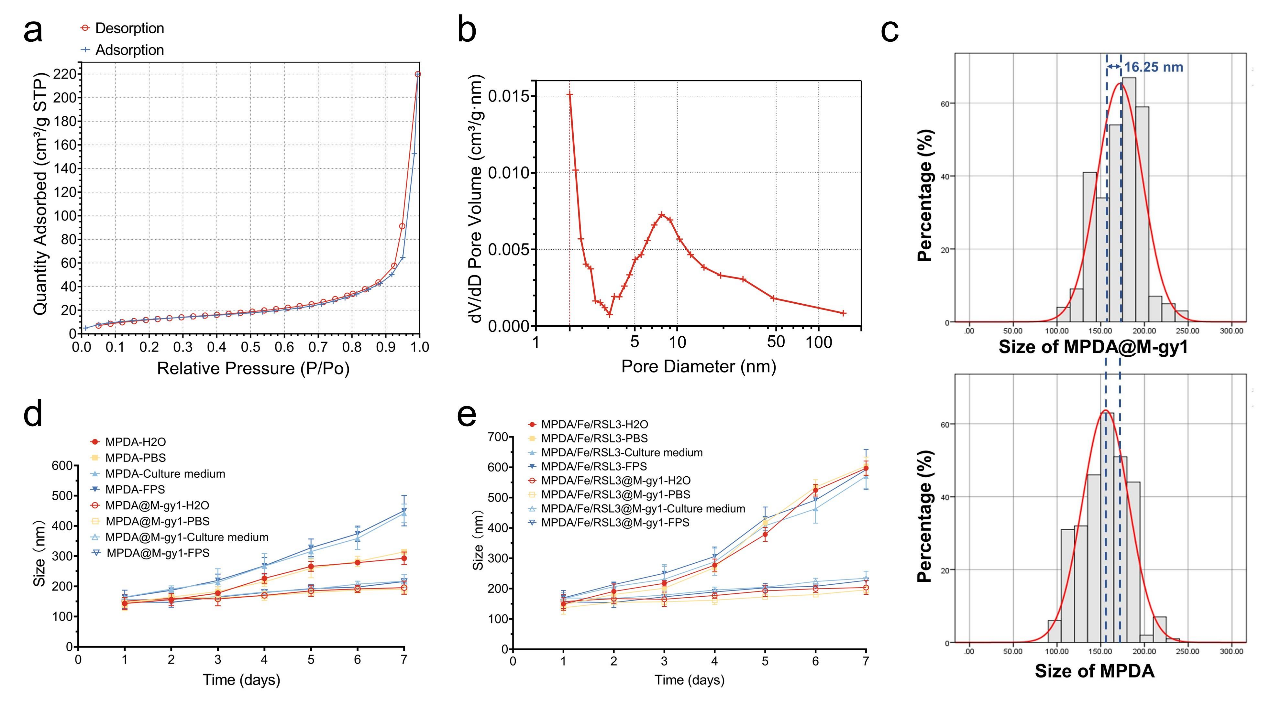


Figure S14 a) Nitrogen adsorption-desorption isotherms curves of MPDA nanoparticles. b) Pore size distribution curve of MPDA nanoparticle. c) Size distribution calculated by transmission electron microscopy (TEM). Size difference between MPDA@M-gy1 and MPDA is consistent with the theoretical thickness of spherical cell membrane. d) Change in size of MPDA and MPDA@M-gy1 in different medium (H_2_O, PBS, culture medium and FPS) for 7 days; n = 3. e) Change in size of MPDA/Fe/RSL3 and MPDA/Fe/RSL3@M-gy1 in different medium (H_2_O, PBS, culture medium and FPS) for 7 days; n = 3.


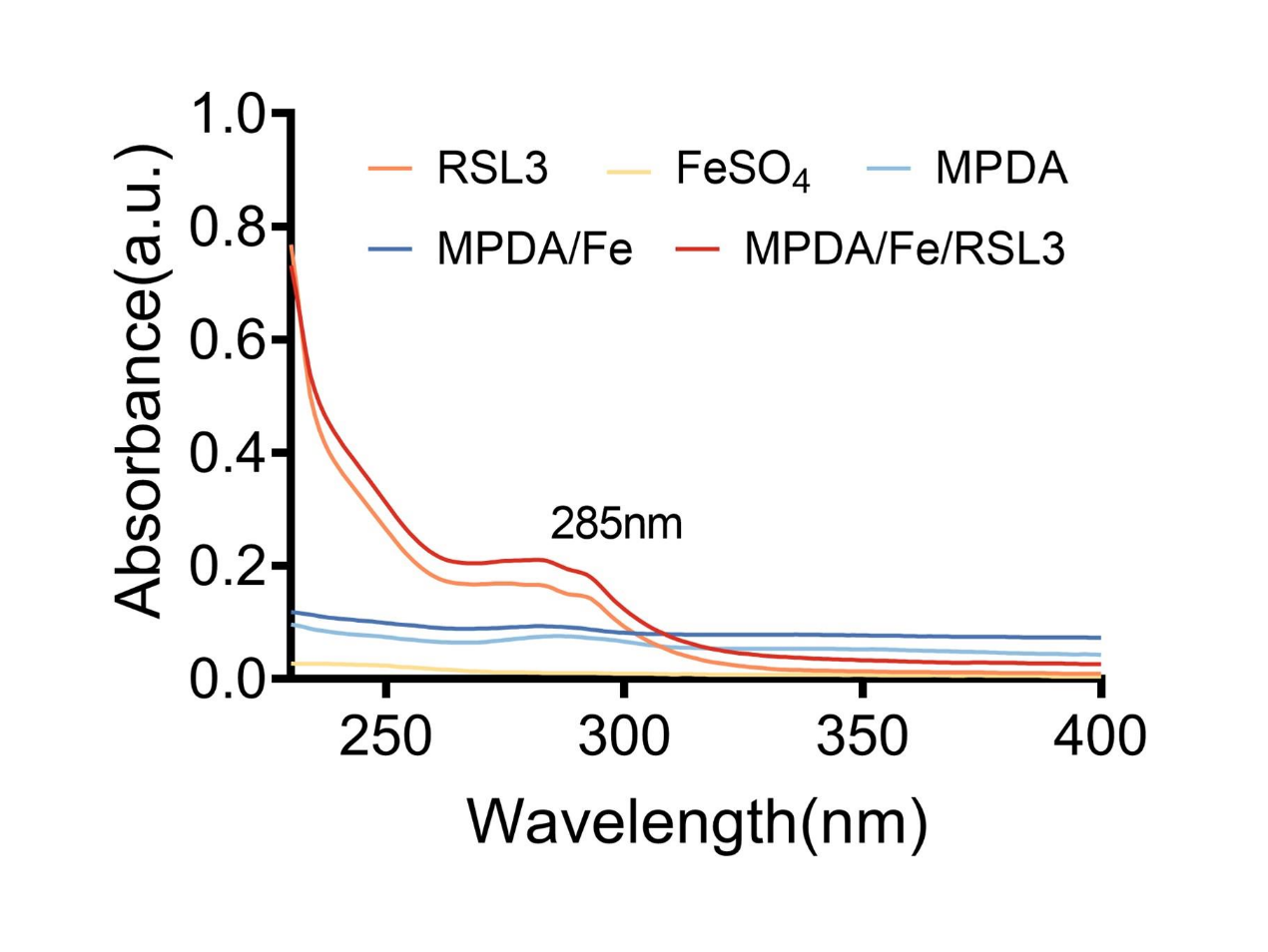


Figure S15 UV-vis spectroscopic analysis of RSL3, FeSO4, MPDA, MPDA/Fe, MPDA/Fe/RSL3.


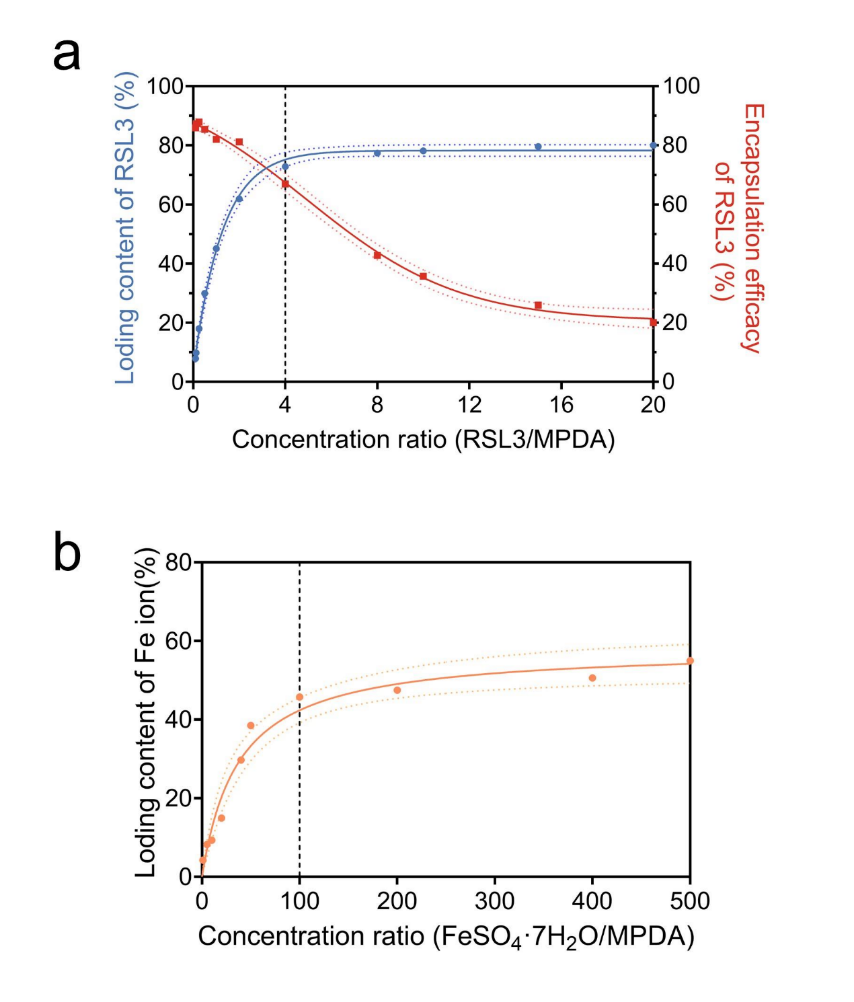


Figure S16 a) RSL3 loading property of MPDA. b) Fe ions loading property of MPDA.


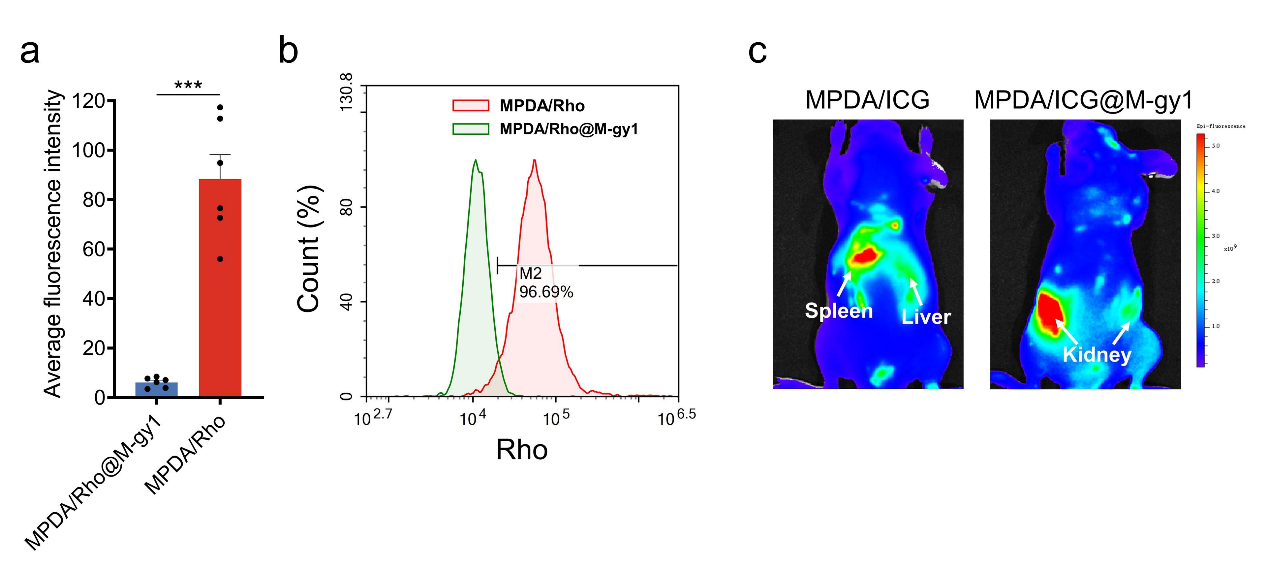
 Figure S17 a) Quantitatively analyzed the average red fluorescence patterns intensity of MPDA/Rho@M-gy1 and MPDA/Rho. b) Flow cytometry histograms of MNPs escaping from Raw264.7-mediated phagocytosis. Rhodamine (Rho) was loaded to MPDA@M-gy1 and MPDA/Rho without TM-expressed gy1 were used as control. c) Fluorescence imaging of normal mice after the intravenous injection of MPDA/ICG or MPDA/ICG@M-gy1. ***p < 0.001.
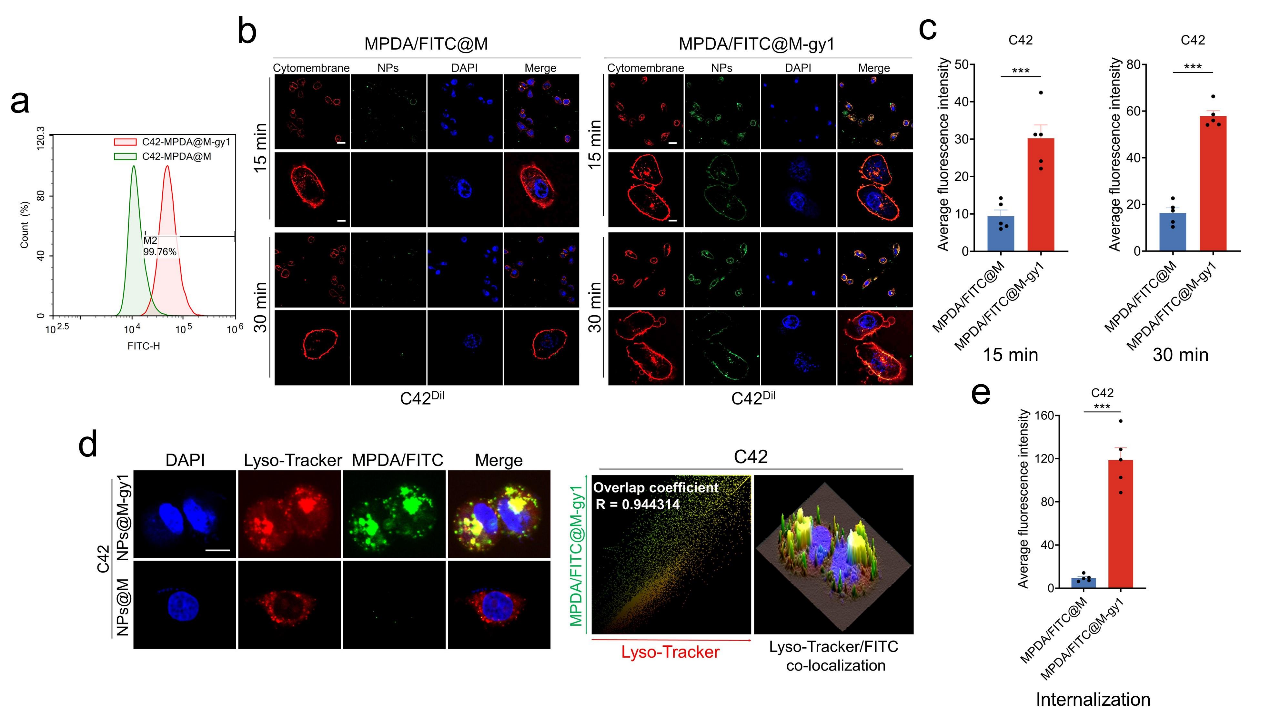


Figure S18 a) Binding efficiency of MNPs to C42 cells detected using flow cytometry. MNPs without TM-expressed gy1 were used as control. b) Specific binding of MNPs to 22RV1 cells evaluated by CLSM. MNPs without TM-expressed gy1 were used as control. MNPs was labeled with FITC (green). Cytomembrane were stained with 1,1′-Dioctadecyl-3,3,3′,3′-Tetramethylindocarbocyanine Perchlorate (Dil) (red). Scale bars: 50 μm and 10 μm. c) Quantitatively analyzed the binding average green fluorescence patterns intensity of MPDA/FITC@M-gy1 and MPDA/FITC@M at time of 15 min and 30 min, respectively. d) Specific internalization of MNPs to C42 cells evaluated by CLSM. MNPs without TM-expressed gy1 cells were used as control. MNPs was labeled with FITC (green). Lysosome were stained with Lyso-Tracker (red). Scale bars: 10 μm. e) Quantitatively analyzed the Internalized average green fluorescence patterns intensity of MPDA/FITC@M-gy1 and MPDA/FITC@M. ***p < 0.001.


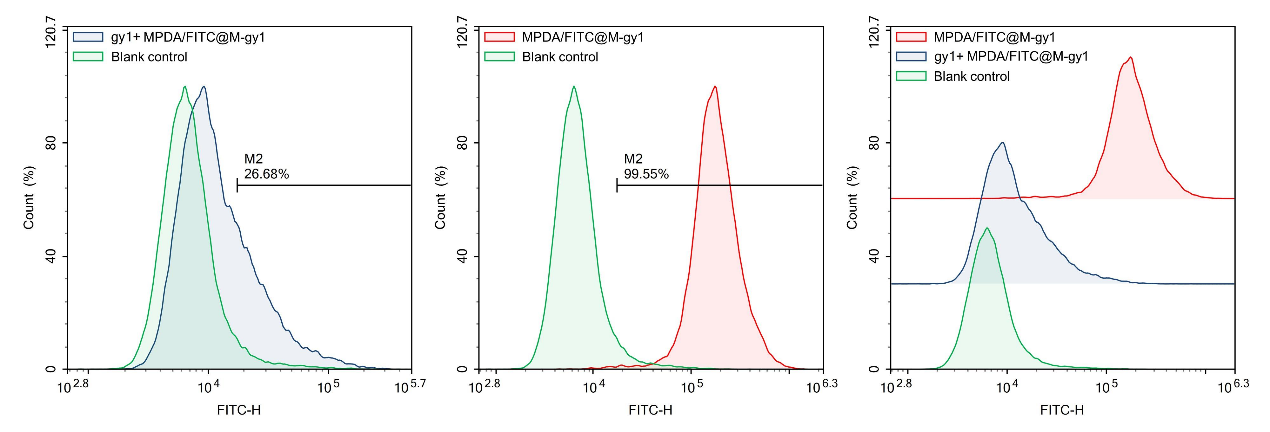


Figure S19 Binding efficiency of MNPs to 22RV1 cells detected through flow cytometry. MNPs co-incubating with 22RV1 cell blocked by excess amount of free gy1 antibody were used as the control. ***p < 0.001.


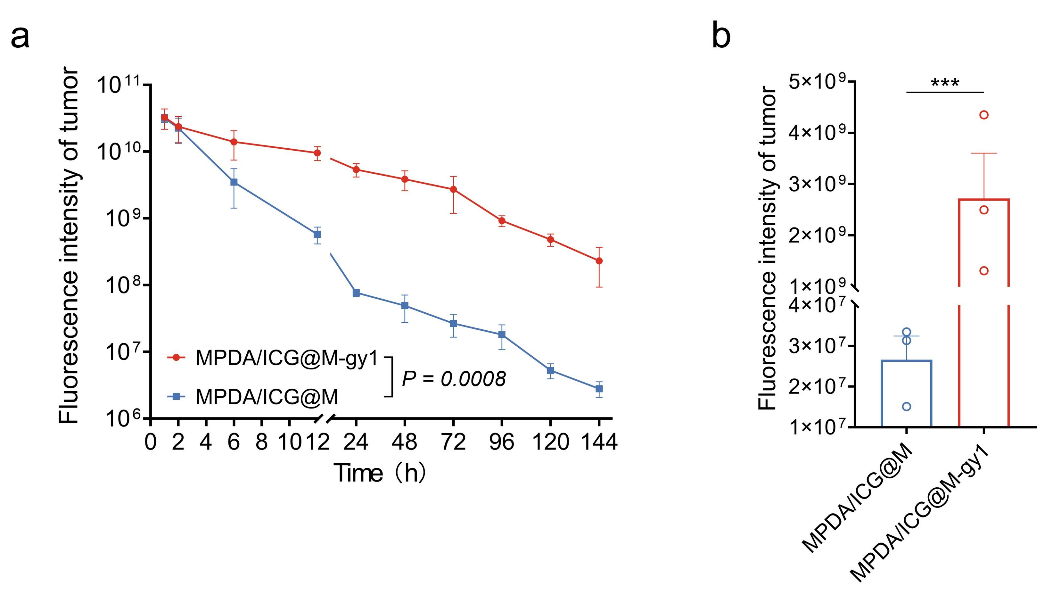


Figure S20 a) Quantitative comparison of ICG fluorescence intensities of subcutaneous tumor sites at different time. b) Quantitative comparison of ICG fluorescence intensity in ex vivo tumors. ***p < 0.001.


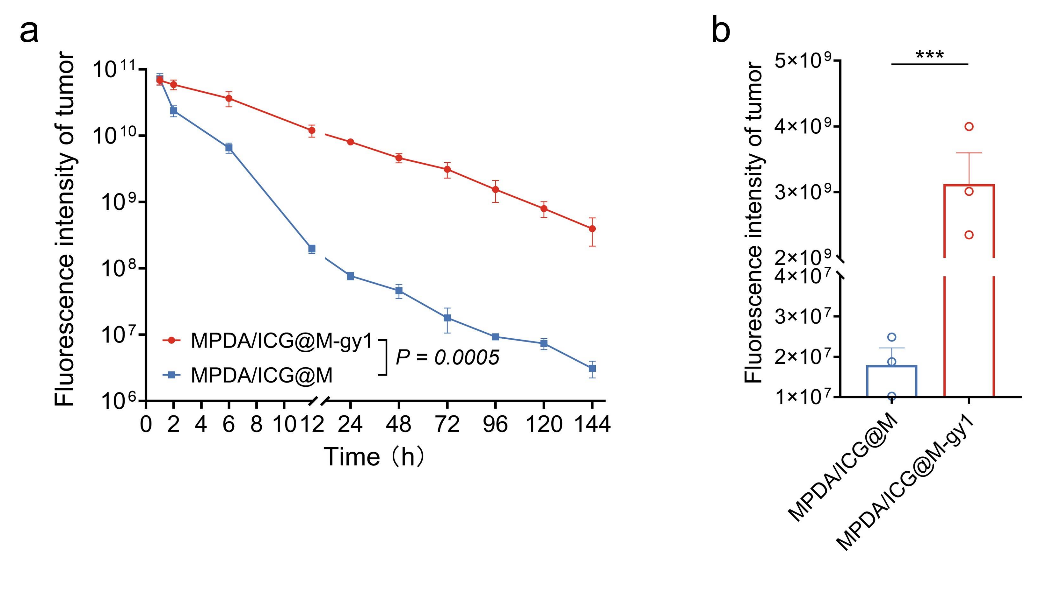


Figure S21 a) Comparison of ICG fluorescence intensities of bone metastasis tumor sites at different time. b) Comparison of ICG fluorescence intensity in ex vivo tumors. ***p < 0.001.


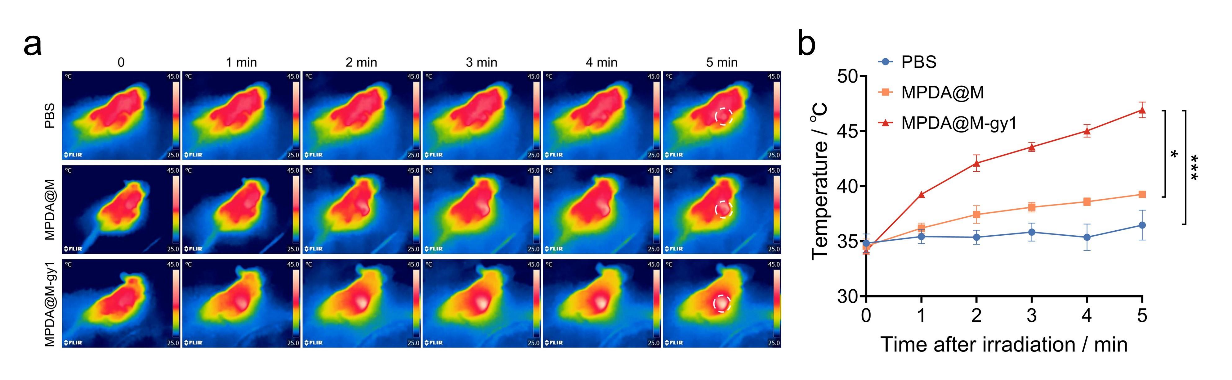


Figure S22 a) IR thermal graphs of 22RV1 intravenous tumor-bearing mice after the intravenous injection with PBS, MPDA@M and MPDA@M-gy1. b) Quantitative comparison of temperature of tumor sites after the intravenous injection with PBS, MPDA@M and MPDA@M-gy1. *p < 0.05. ***p < 0.001.


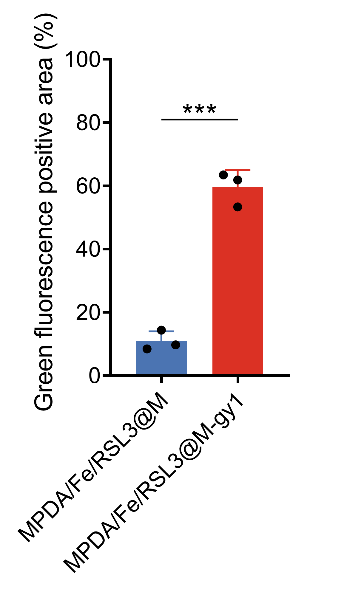


Figure S23 Quantitatively analyzed the average green fluorescence positive area of MPDA/FITC@M-gy1 and MPDA/FITC@M in the internal tumor hypovascular zone. ***p < 0.001.


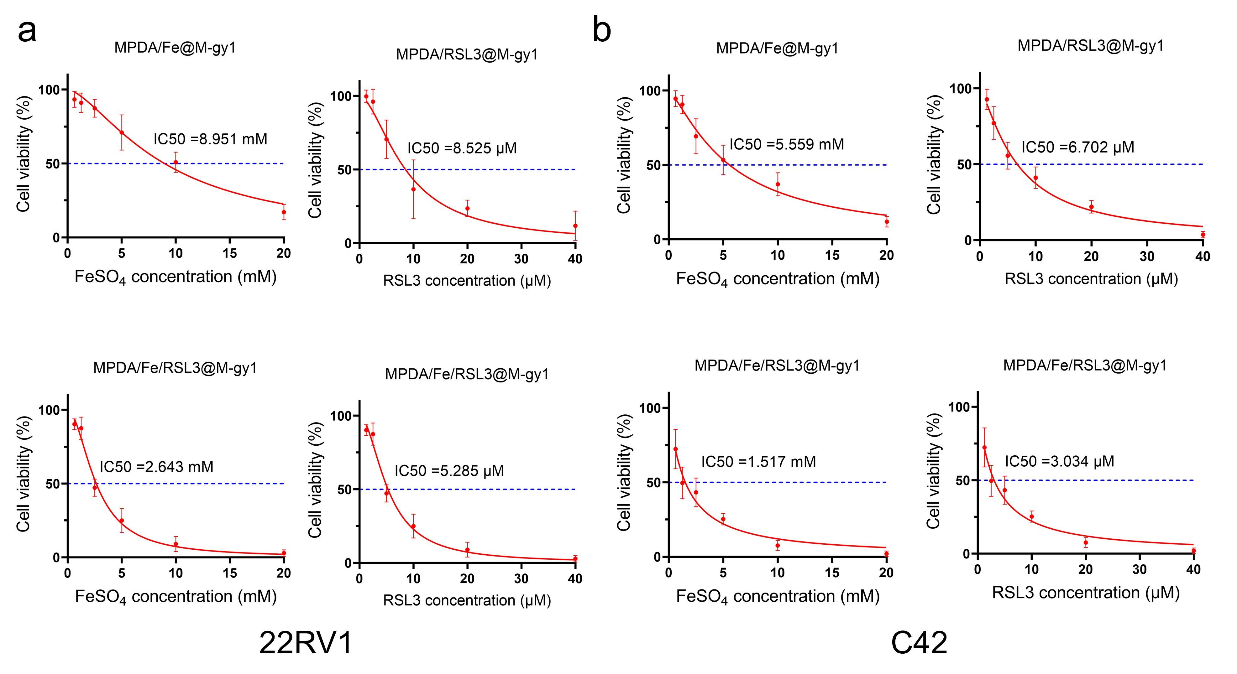


Figure S24 Cell viability and IC50 of different cell lines following MNPs treatment for 72 hours. a) 22RV1 cell line. b) C42 cell line.


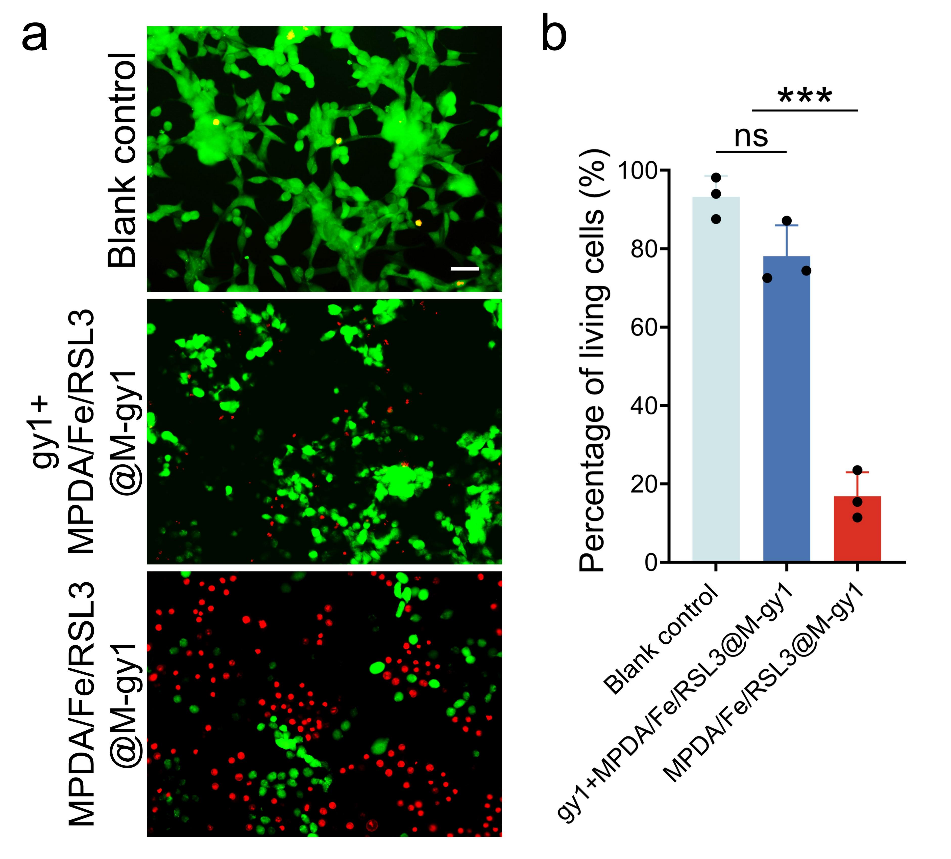


Figure S25 a) Live–dead cell imaging for surviving 22RV1 cells after incubation with different agents. Scale bar: 50 μm. b) Quantitative analysis of the percentage of living cells. ns represented no significance, ***p < 0.001.


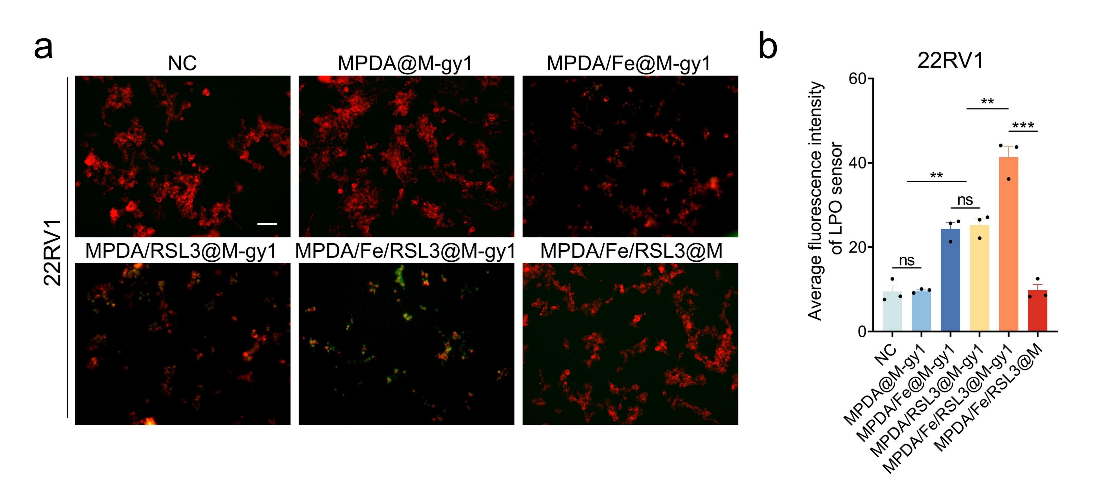


Figure S26 a) CLSM monitoring the intracellular lipoperoxide accumulation on the 22RV1 cells after incubation with different groups. Scale bars: 50 μm. b) Quantitatively analyzed the average fluorescence intensity of LPO sensor in 22RV1 cells. ns represented no significance, **p < 0.01, ***p < 0.001.


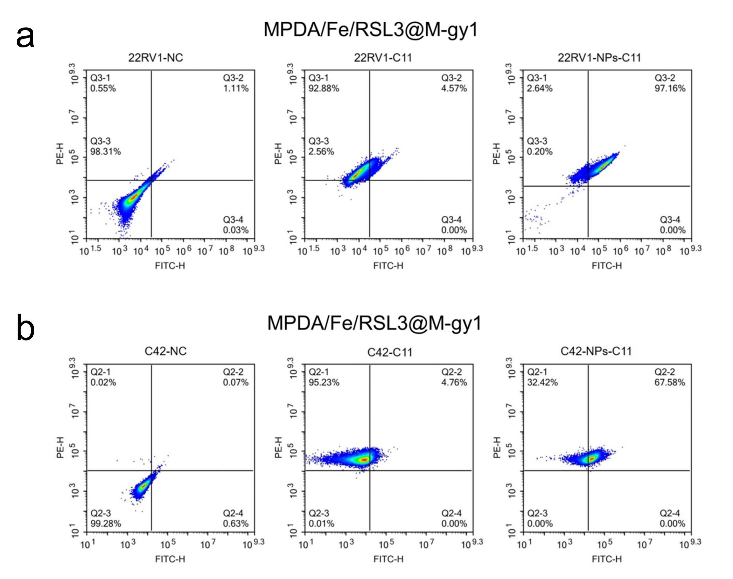


Figure S27 The change of LPO levels (BODIPY 581/591 C11 probe) in cells treated with MPDA /Fe/RSL3@M-gy1 treatment were determined by Flow cytometry. a) 22RV1 cell line. b) C42 cell line.


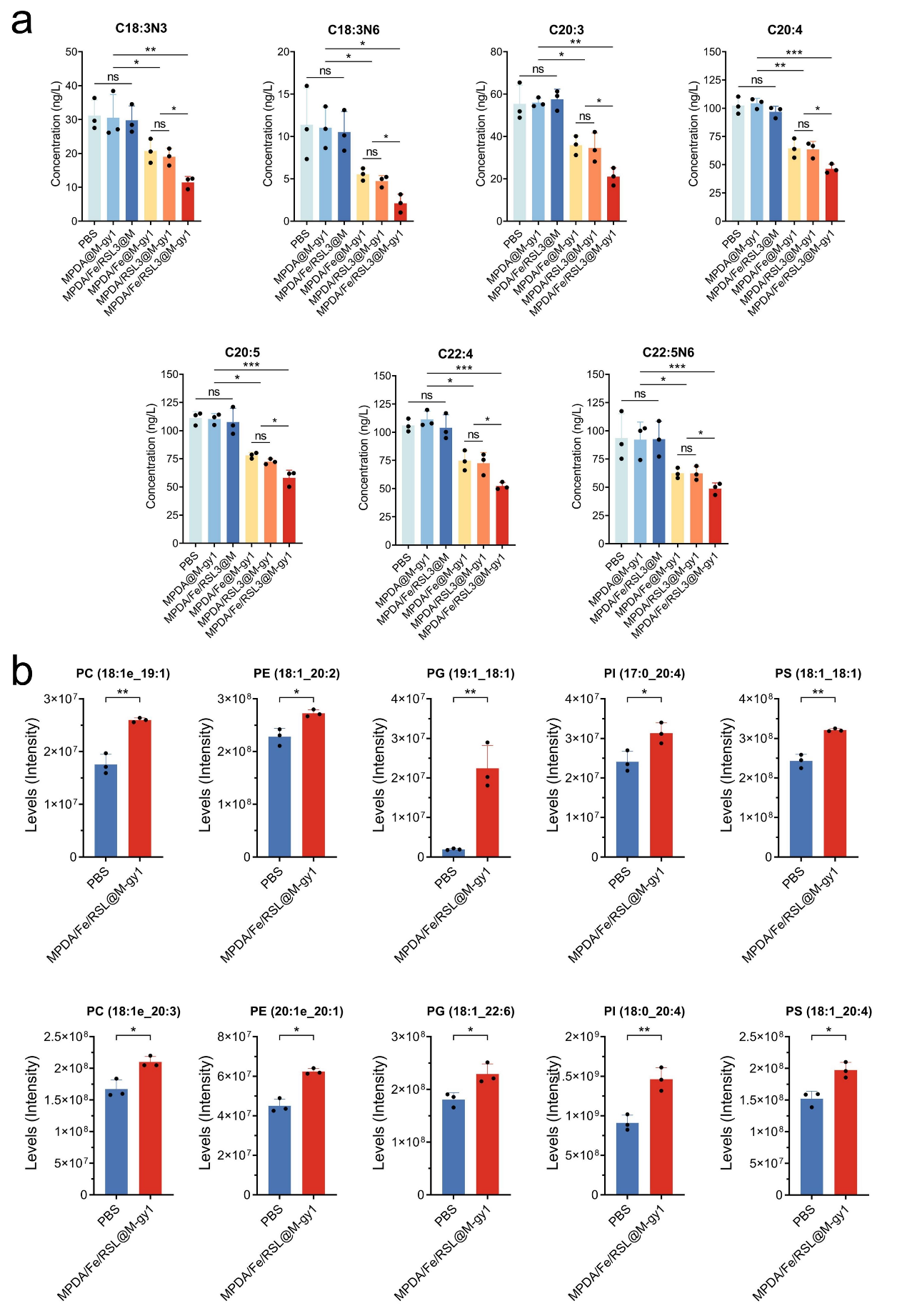


Figure S28 a) Levels of selected PUFAs as the substrates for peroxidation in 22RV1 cells of each treatment group. b) Levels of selected PL-PUFAs with the indicated treatment. ns represented no significance, *p< 0.05, **p < 0.01, ***p < 0.001.


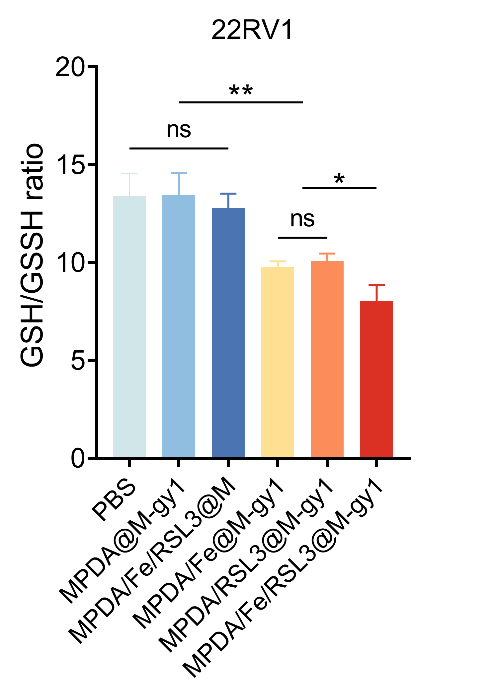


Figure S29 GSH/GSSH ratio in 22RV1 cells after incubation with different agents. ns represented no significance, *p< 0.05, **p < 0.01.


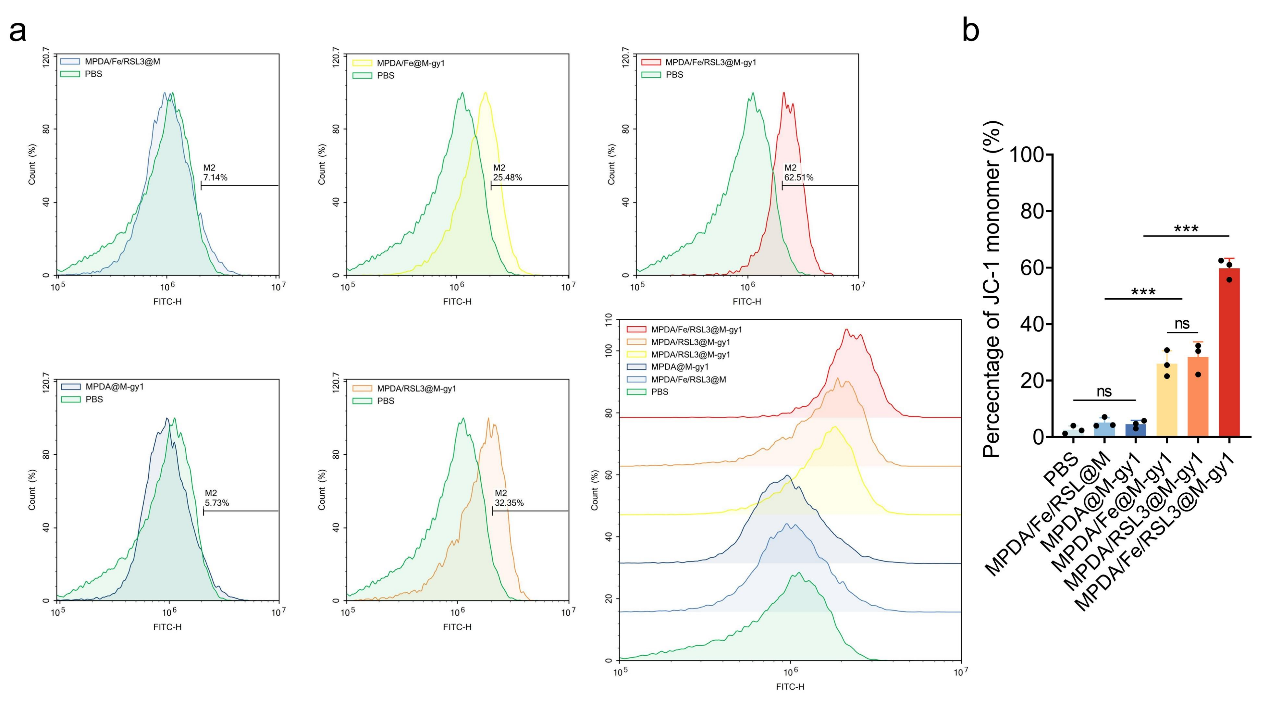


Figure S30 a) Flow cytometry analysis of mitochondrial membrane potential in different agent–treated 22RV1 cells by using a JC-1 fluorescent probe. b) Quantitatively analyzed the percentage of JC-1 monomer in 22RV1 cells. ns represented no significance, ***p < 0.001.


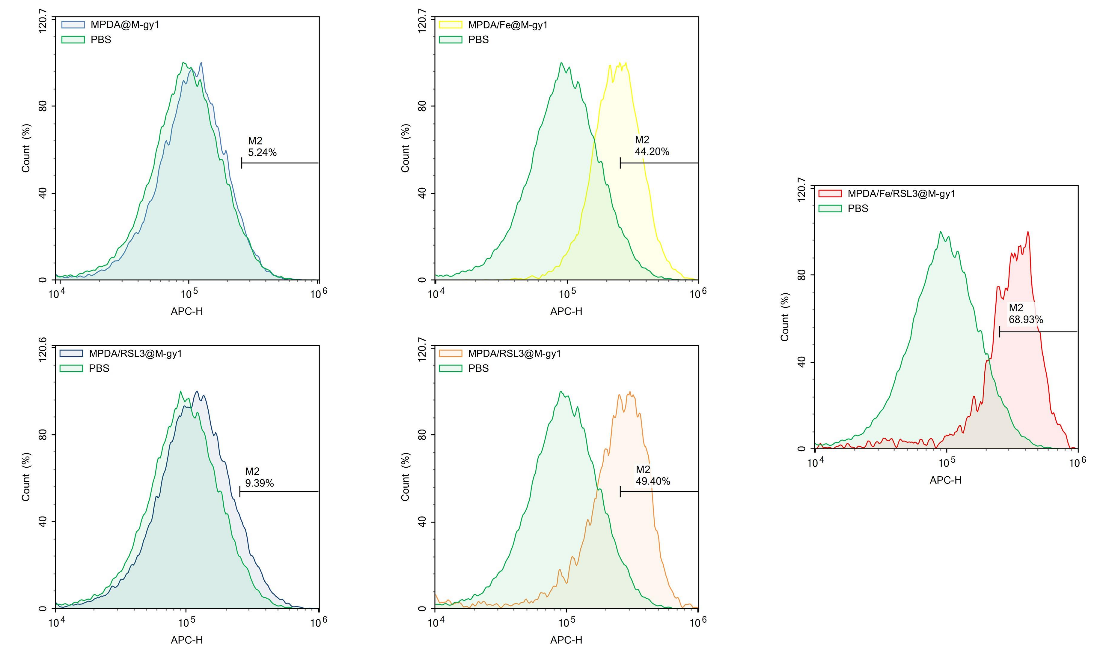


Figure S31 Flow cytometry analysis of ROS levels in different agent–treated 22RV1 cells by using a ROS fluorescent probe (CellROX Deep Red probe).


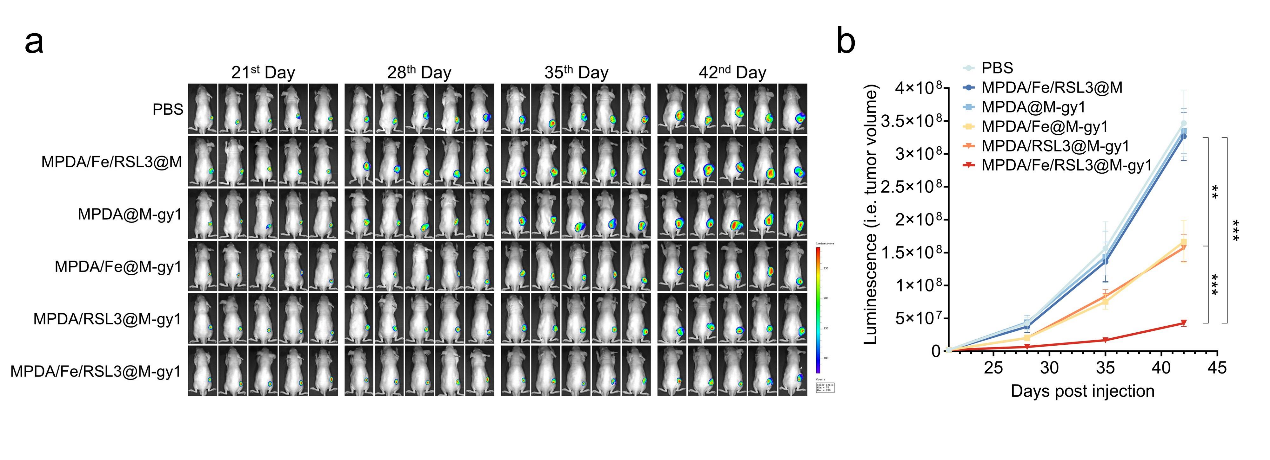


Figure S32 a) In vivo bioluminescence images of subcutaneous tumor-bearing mice in different groups; n = 5. b) Quantification of bioluminescence intensity. **p < 0.01, ***p < 0.001.


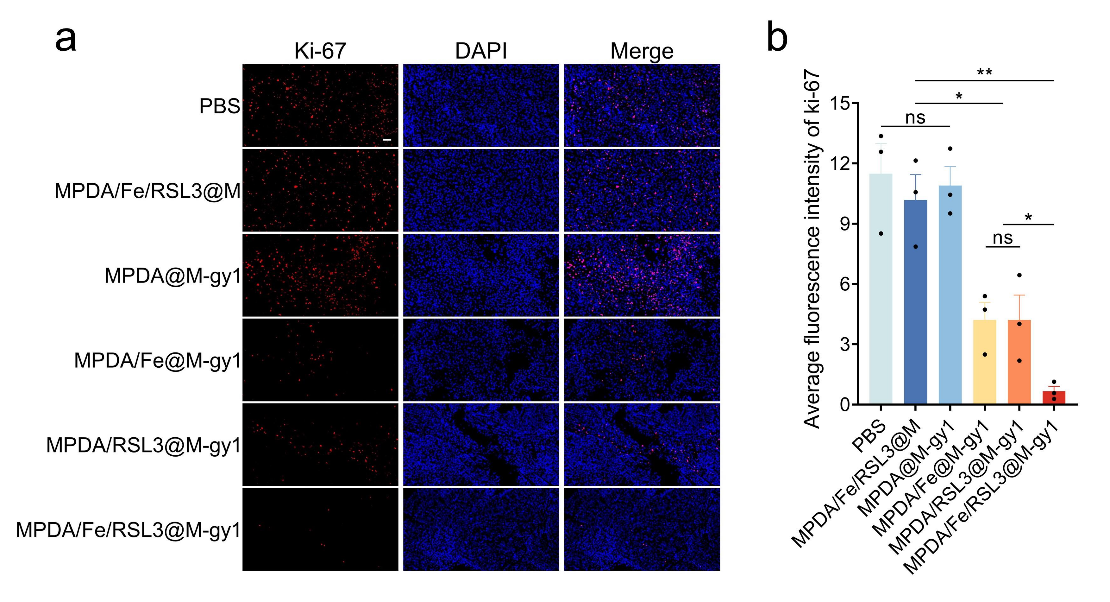


Figure S33 a) Immunofluorescence images of ki-67 expression in 22RV1 tumor tissues after the 21-day treatment period of different groups. Scale bars: 50 μm. b) Quantitatively analyzed average fluorescence intensity of ki-67 in different groups. ns represented no significance, *p < 0.05, **p < 0.01.


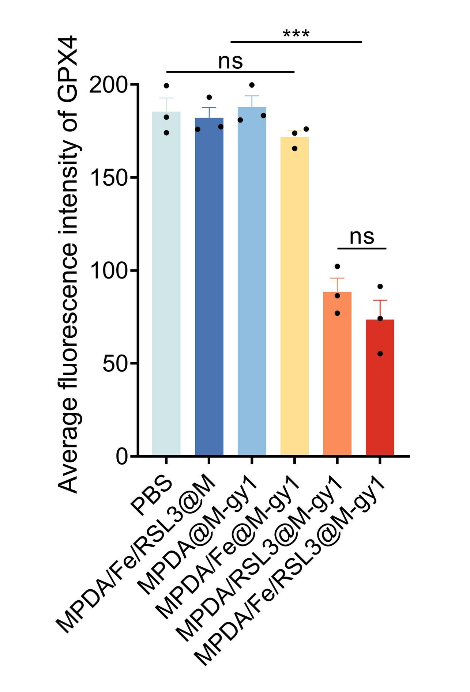


Figure S34 Quantitatively analyzed average fluorescence intensity of GPX4 in different groups. ns represented no significance, *p < 0.05, ***p < 0.001.


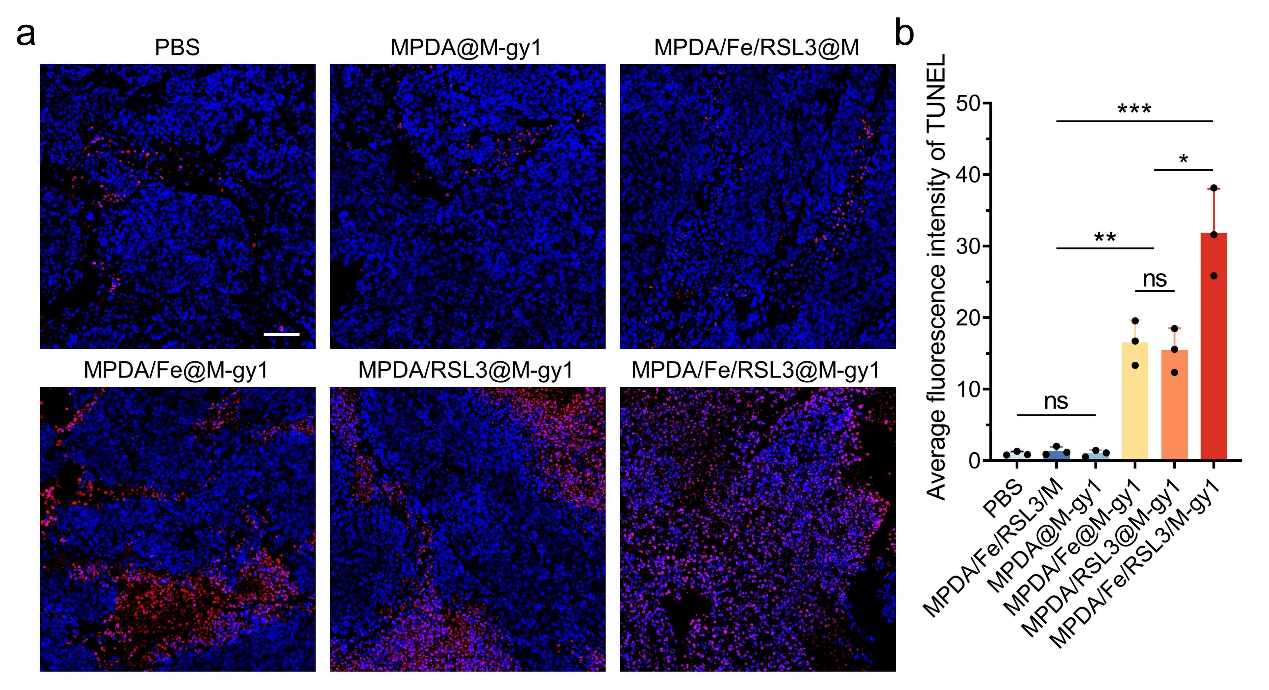


Figure S35 a) Immunofluorescence images of TUNEL expression in 22RV1 tumor tissues after the 21-day treatment period of different groups. Scale bars: 50 μm. b) Quantitatively analyzed average fluorescence intensity of TUNEL in different groups. ns represented no significance, *p < 0.05, **p < 0.01, ***p < 0.01.


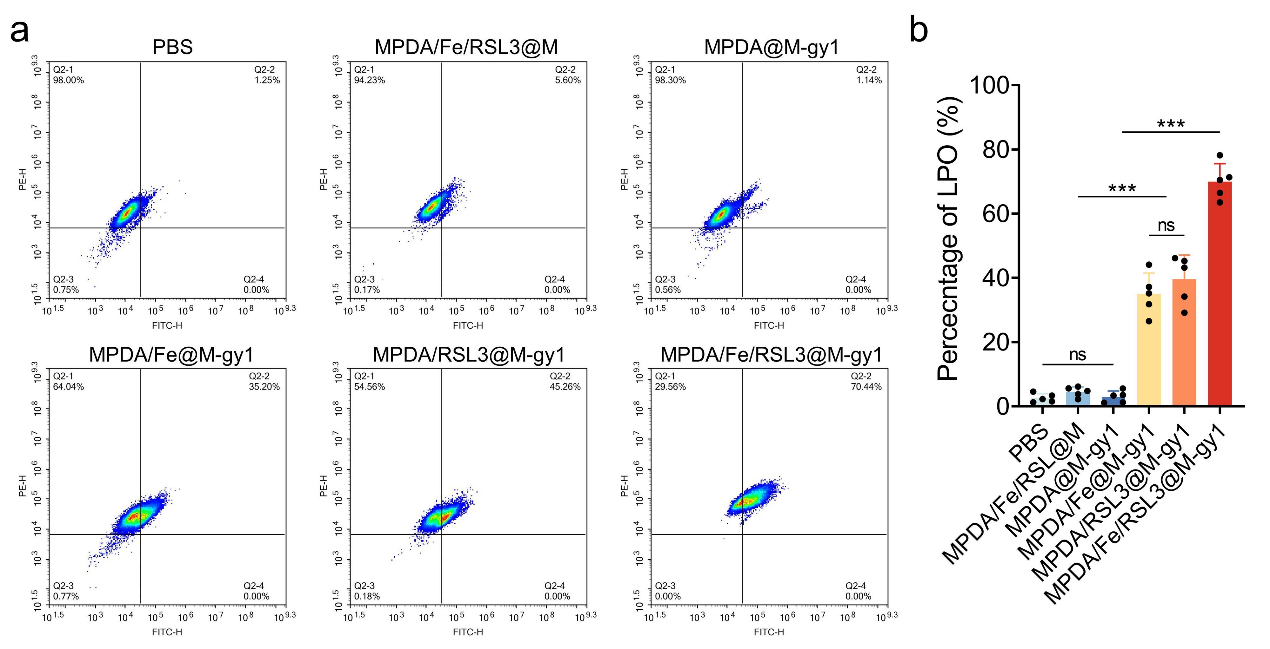


Figure S36 The change of LPO levels (BODIPY 581/591 C11 probe) in primary cells of 22RV1 tumor tissues treated with different groups were determined by Flow cytometry. b) Quantitatively analyzed the percentage of LPO in 22RV1 cells. ns represented no significance, ***p < 0.001.


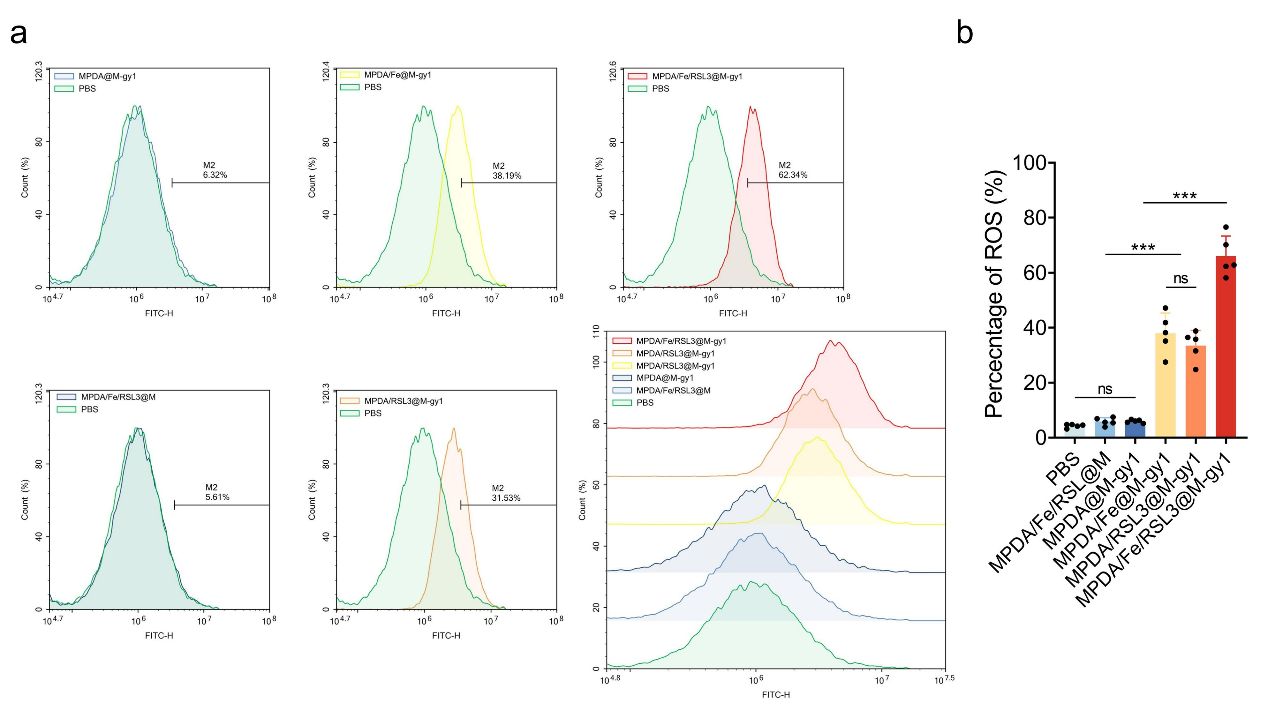


Figure S37 The change of ROS levels (CellROX Green probe) in primary cells of 22RV1 tumor tissues treated with different groups were determined by Flow cytometry. b) Quantitatively analyzed the percentage of ROS in 22RV1 cells. ns represented no significance, ***p < 0.001.


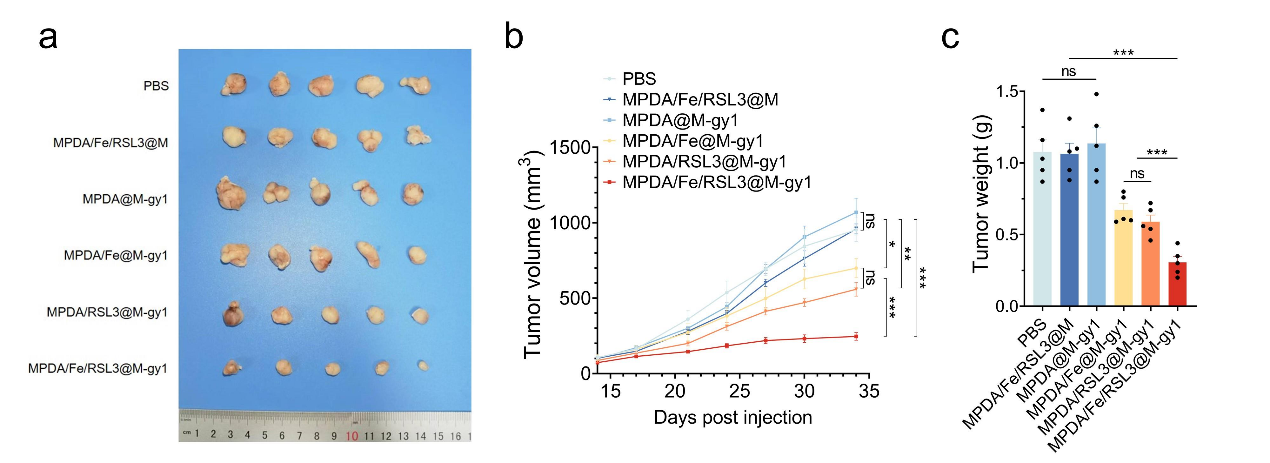


Figure S38 a) Visual comparison of whole tumors from MyC-CaP^psma+^ tumor-bearing FVB mice after treatment in different groups. b) Changes in tumor volumes in MyC-CaP^psma+^ tumor-bearing FVB mice after different treatments (n = 5). c) Visual comparison of whole tumors from MyC-CaP^psma+^ tumor-bearing FVB mice after treatment in different groups. ns, no significance. *p < 0.05, **p < 0.01, ***p < 0.001.


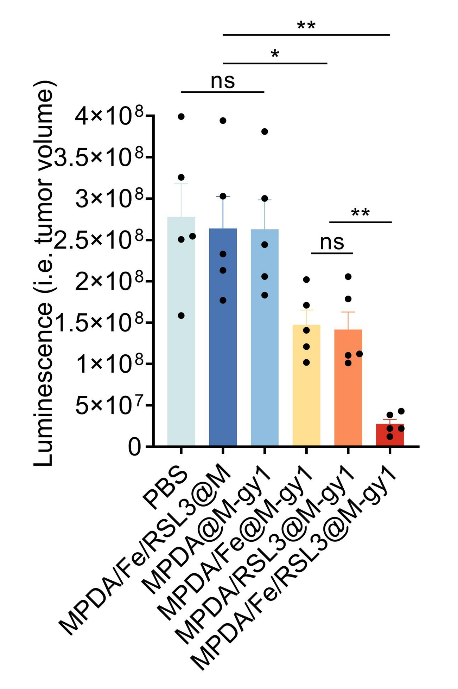


Figure S39 Quantification of bioluminescence intensity after the last treatment of bone metastasis mice in different groups; n = 5. ns represented no significance, *p < 0.05, **p < 0.01.


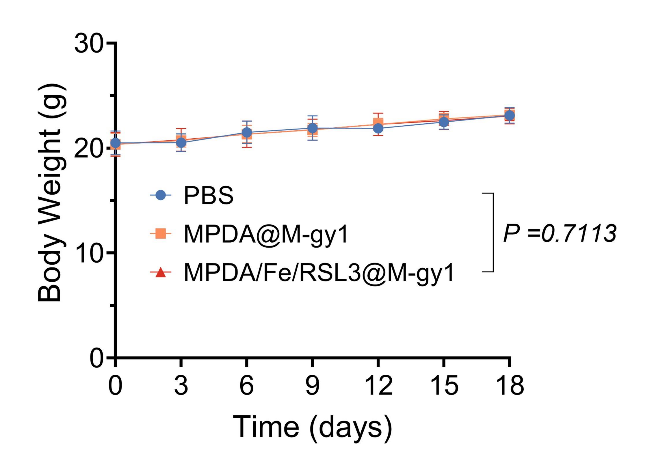


Figure S40 Quantitative comparison of mice weight of normal BALB/c nude mice after the intravenous injection with PBS, MPDA@M-gy1 and MPDA/Fe/RSL3@M-gy1 for 18 days.


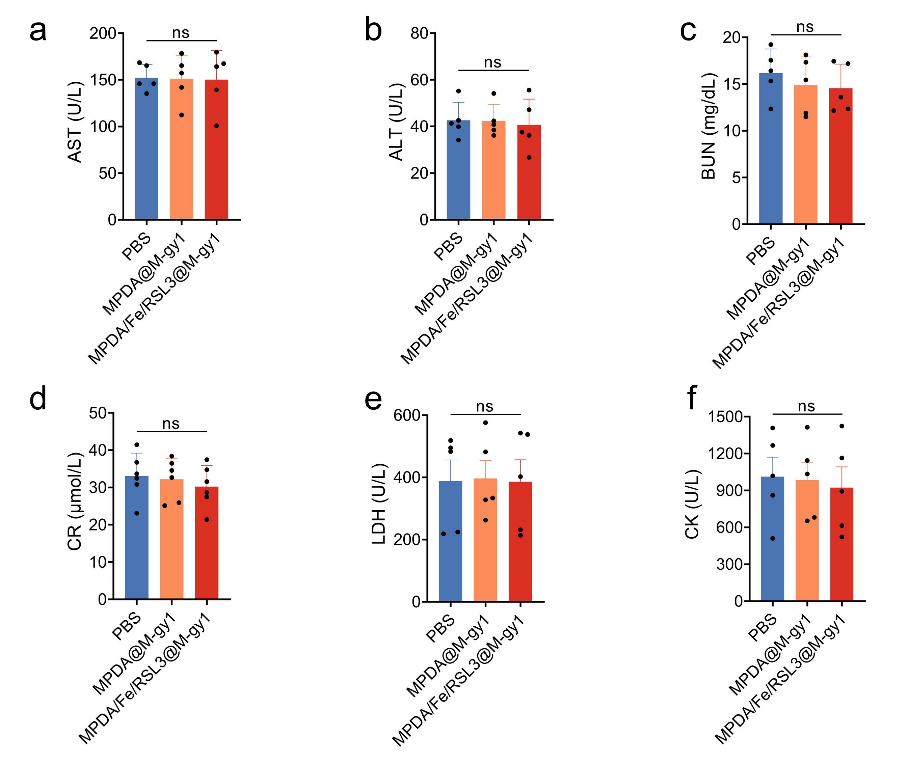


Figure S41 Serological detection of liver, kidney and myocardium in different groups. Alanine aminotransferase (ALT) and aspartate transaminase (AST) are commonly used index evaluating liver function. Blood urea nitrogen (BUN) and serum creatinine (CR) are commonly used index evaluating kidney function. Creatine kinase (CK) and lactate dehydrogenase (LDH) are commonly used index evaluating myocardial function. ns represented no significance.


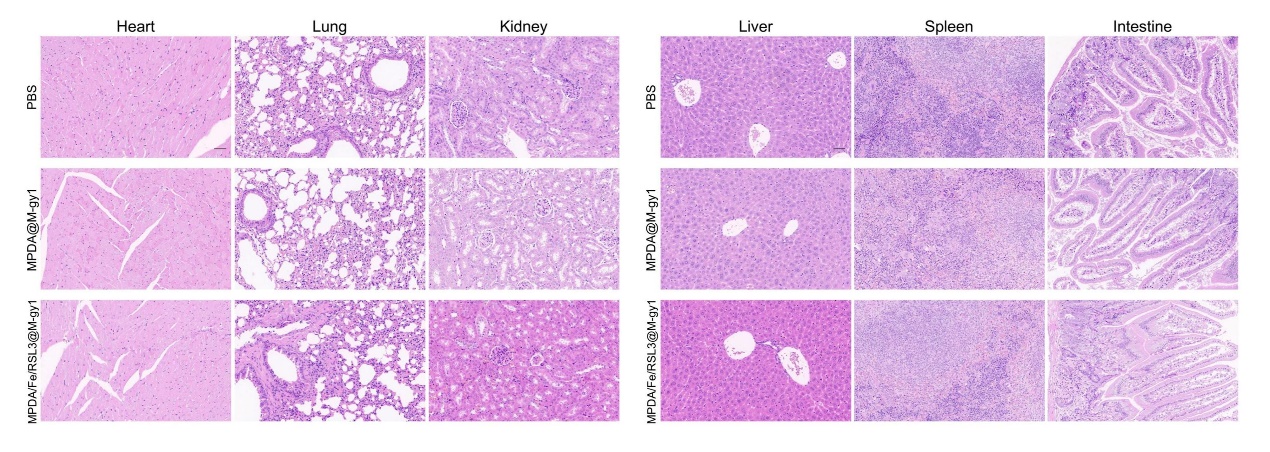


Figure S42 Peripheral organ-toxicity of different groups evaluated by H&E staining. Scale bars: 50 μm.


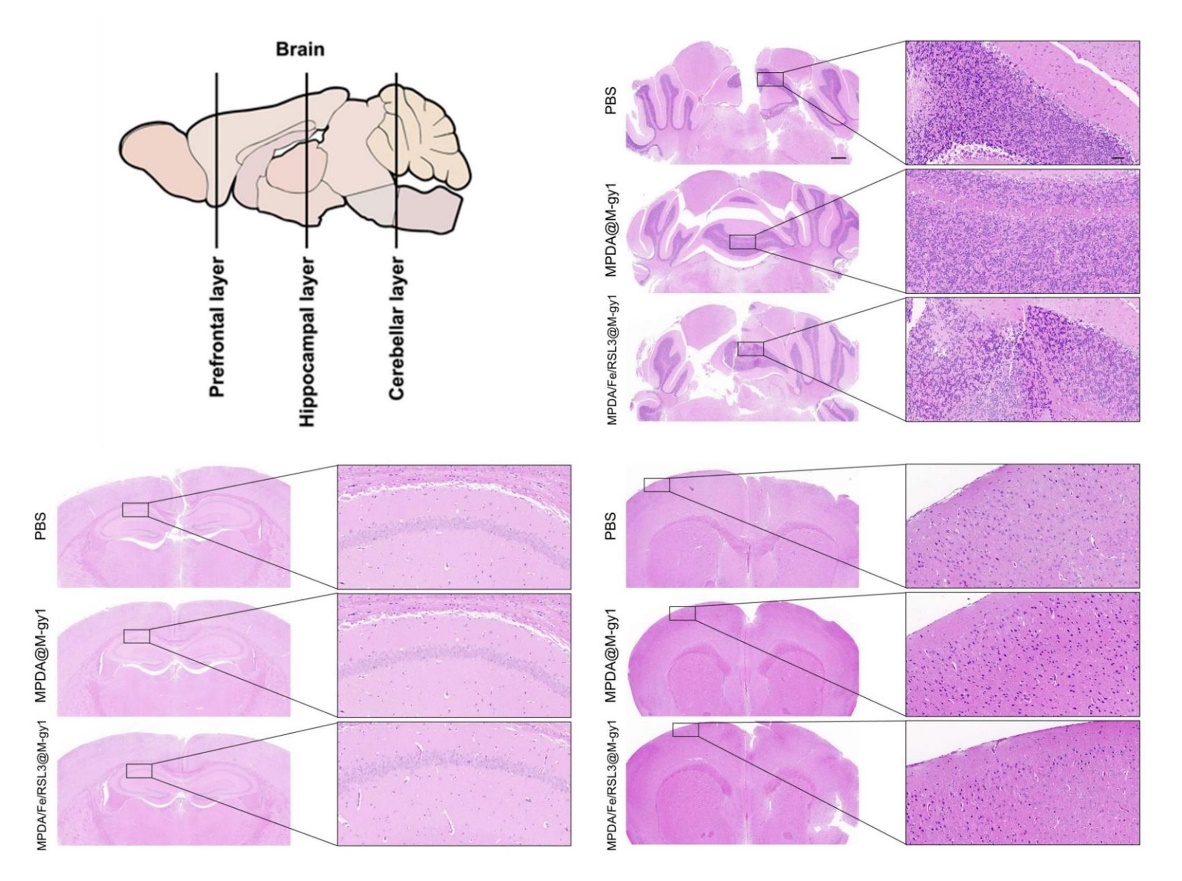


Figure S43 Neurotoxicity of different groups evaluated by H&E staining. Scale bars: 2000 μm and 100 μm.
